# *Albugo*-imposed changes to tryptophan-derived antimicrobial metabolite biosynthesis may contribute to suppression of non-host resistance to *Phytophthora infestans* in *Arabidopsis thaliana*

**DOI:** 10.1186/s12915-017-0360-z

**Published:** 2017-03-20

**Authors:** David C. Prince, Ghanasyam Rallapalli, Deyang Xu, Henk-jan Schoonbeek, Volkan Çevik, Shuta Asai, Eric Kemen, Neftaly Cruz-Mireles, Ariane Kemen, Khaoula Belhaj, Sebastian Schornack, Sophien Kamoun, Eric B. Holub, Barbara A. Halkier, Jonathan D. G. Jones

**Affiliations:** 1 The Sainsbury Laboratory, Norwich Research Park, Norwich, NR4 7UH United Kingdom; 2 School of Biological Sciences, University of East Anglia, Norwich Research Park, Norwich, United Kingdom; 3 Norwich Medical School, University of East Anglia, Norwich Research Park, Norwich, UK; 4 DynaMo Center, Department of Plant and Environmental Sciences, Faculty of Science, University of Copenhagen, 40 Thorvaldsensvej, DK-1871 Frederiksberg C, Denmark; 5 Department of Crop Genetics, John Innes Centre, Norwich Research Park, Norwich, NR4 7UH UK; 6 Department of Biology and Biochemistry, University of Bath, Bath, UK; 7 Plant Immunity Research Group, Center for Sustainable Resource Science, RIKEN Yokohama Institute, Yokohama, Japan; 8 Max Planck Research Group Fungal Biodiversity, Max Planck Institute for Plant Breeding Research, Cologne, Germany; 9 Sainsbury Laboratory, University of Cambridge, Cambridge, UK; 10 School of Life Sciences, Warwick Crop Centre, University of Warwick, Wellesbourne, UK

**Keywords:** *Phytophthora infestans*, *Albugo*, *Arabidopsis thaliana*, Glucosinolates, Camalexin, Salicylic acid, Non-host resistance

## Abstract

**Background:**

Plants are exposed to diverse pathogens and pests, yet most plants are resistant to most plant pathogens. Non-host resistance describes the ability of all members of a plant species to successfully prevent colonization by any given member of a pathogen species. White blister rust caused by *Albugo* species can overcome non-host resistance and enable secondary infection and reproduction of usually non-virulent pathogens, including the potato late blight pathogen *Phytophthora infestans* on *Arabidopsis thaliana*. However, the molecular basis of host defense suppression in this complex plant–microbe interaction is unclear. Here, we investigate specific defense mechanisms in Arabidopsis that are suppressed by *Albugo* infection.

**Results:**

Gene expression profiling revealed that two species of *Albugo* upregulate genes associated with tryptophan-derived antimicrobial metabolites in Arabidopsis. *Albugo laibachii-*infected tissue has altered levels of these metabolites, with lower indol-3-yl methylglucosinolate and higher camalexin accumulation than uninfected tissue. We investigated the contribution of these *Albugo*-imposed phenotypes to suppression of non-host resistance to *P. infestans*. Absence of tryptophan-derived antimicrobial compounds enables *P. infestans* colonization of Arabidopsis, although to a lesser extent than *Albugo*-infected tissue. *A. laibachii* also suppresses a subset of genes regulated by salicylic acid; however, salicylic acid plays only a minor role in non-host resistance to *P. infestans*.

**Conclusions:**

*Albugo* sp. alter tryptophan-derived metabolites and suppress elements of the responses to salicylic acid in Arabidopsis. *Albugo* sp. imposed alterations in tryptophan-derived metabolites may play a role in Arabidopsis non-host resistance to *P. infestans*. Understanding the basis of non-host resistance to pathogens such as *P. infestans* could assist in development of strategies to elevate food security.

**Electronic supplementary material:**

The online version of this article (doi:10.1186/s12915-017-0360-z) contains supplementary material, which is available to authorized users.

## Background

Plants are exposed to diverse pathogens and pests, yet most plants are resistant to most plant pathogens. Successful pathogens and pests suppress plant immunity to enable plant colonization. Current models envisage a multi-level evolutionary arms race between plants and pathogens or pests [1–4]. Plant defense responses are initiated by recognition of pathogen or pest attack via detection of pathogen molecules by plant cell surface receptors. Relatively invariant and indispensable molecules known as microbe- or pathogen-associated molecular patterns, are recognized by transmembrane pattern recognition receptors at the plasma membrane. This leads to signaling responses that result in pattern-triggered immunity (PTI). PTI is sufficient to prevent colonization by most non-adapted pathogens or pests, but pathogens which are adapted to particular host plants have evolved effectors that suppress PTI. In turn, plants evolved intracellular receptors that recognize the structure or action of effectors, resulting in effector-triggered immunity (ETI). The pathogen may subsequently adapt to the host further by evolving a variant non-recognized effector or evolving other effectors to suppress ETI.

Non-host resistance (NHR) describes the ability of all members of a plant species to successfully prevent colonization by any given member of a pathogen species [5, 6]. In principle, NHR might result from the triggering of PTI, ETI or antimicrobial secondary metabolites. It has been proposed that the more distantly related a non-host plant is from a host plant for a pathogen, the greater the relative contribution of PTI compared to ETI in NHR [7].

*Albugo* species are obligate biotrophic oomycetes that cause white blister or white rust disease in plants [8]. *Albugo laibachii* specializes on Arabidopsis [9], whereas *A. candida* is comprised of physiological races (*formae speciales*) that cause disease in diverse members of the *Brassicaceae*, *Cleomaceae*, and *Capparaceae* [8, 10]. Although most plants resist most pathogens, *Albugo* spp. not only overcome plant immune responses against themselves, but also suppress immunity against other filamentous pathogens. *A. laibachii* and *A. candida* can suppress resistance in Arabidopsis and *Brassica juncea* to downy mildews and other filamentous pathogens to which the plants are naturally resistant [10, 11]. Suppression of immunity could allow *A. candida* strains with different host ranges to co-exist on the same host and sexually reproduce, thus allowing genetic exchange that potentially facilitates colonization of new hosts [10].

We recently found that *A. laibachii* suppresses Arabidopsis non-host resistance to *Phytophthora infestans* [12]. *P. infestans* is a hemibiotrophic oomycete that causes late blight disease in potato and tomato, leading to global yield losses [13], and is adapted to a few solanaceous plant species [14], but not to Arabidopsis [15]. A better understanding of the mechanisms that prevent *P. infestans* colonizing Arabidopsis may lead to new methods for controlling late blight disease in crop species. Crop protection strategies based on non-host resistance are of interest because they have the potential to be durable. Initial efforts to understand Arabidopsis NHR to *P. infestans* examined cytological and gene expression responses. Resistance is associated with epidermal cell death and induction of jasmonic acid (JA) responses followed by salicylic acid (SA) responses [15, 16]. However, the *coronatine-insensitive 1* (*coi-1*) mutant, compromised in JA signaling, is resistant to *P. infestans* [17]. Subsequently, several Arabidopsis genes involved in NHR to *P. infestans* have been identified. *Penetration2* (*PEN2*) encodes an atypical myrosinase that hydrolyses 4-methoxyindol-3-ylmethylglucosinolate (4MO-I3M) into antimicrobial compounds [18]. *PEN3* encodes a pleiotropic drug resistance ATP-binding cassette (ABC) transporter implicated in secreting antimicrobial compounds, including those produced by PEN2 [19–21]. *pen2* and *pen3*/*atpdr8* mutants show increased epidermal penetration and invasive growth by *P. infestans* and subsequent enhanced plant cell death in response [19, 22, 23]. A forward genetic screen to identify additional components of Arabidopsis NHR to *P. infestans* identified *enhanced response to Phytophthora* (*erp*) mutants [24, 25]. *erp1* encodes a phospholipid:sterol acyltransferase and shows increased cell death and callose depositions in the mesophyll without increased growth by the pathogen [24]. e*rp6* encodes EDR1 (enhanced disease resistance1) and plays a role in post-invasive NHR to *P. infestans*, where it acts as a negative regulator of PTI, SA signaling, and callose deposition [25]. However, while *P. infestans* can penetrate into the leaf tissue of some of the Arabidopsis mutants so far identified, there have been no reports of *P. infestans* producing haustoria or sporulating.

Compounds that are not directly involved in the primary processes of basic growth and development are termed secondary metabolites, which comprise a large collection of diverse small molecules. Specific classes of secondary metabolite are often restricted to a narrow phylogenetic lineage [26], but may perform conserved functions in plant immunity [27]. Arabidopsis secondary metabolites with a role in defense include the tryptophan-derived secondary metabolites glucosinolates, which are mostly restricted to the order Brassicales [28], and camalexin that appears to be present only in species belonging to the Camelinae tribe [29]. Camalexin and indolic glucosinolates play a role in plant immunity against diverse microbial pathogens and insect pests (reviewed by [30]). Interestingly, tryptophan-derived secondary metabolites have recently been shown to play a role in immunity to the oomycetes *Phytophthora brassicae* and *Phytophthora capsici* [31, 32]. The importance of camalexin to plant immunity in the Brassicales can also be seen from the examples of pathogens that detoxify these compounds in order to colonize the host [33–35].

The phenolic phytohormone SA plays an important signaling role in plant immunity [36]. SA regulates immunity, especially against biotrophs and hemibiotroph pathogens [37]. PTI and ETI lead to the accumulation of SA [38–40] and therefore the combined effects can be thought of as SA-triggered immunity (SATI). Mutants in SA signaling are more susceptible to both adapted and non-adapted pathogens (e.g. [31, 41, 42]), and effectors from several pathogen species target SA accumulation and SATI (reviewed by [43]).

The *Albugo-*Arabidopsis pathosystem offers the opportunity to investigate the mechanistic nature of immune-suppression in detail. We investigated how *Albugo* spp. suppress Arabidopsis NHR to *P. infestans*. We used expression profiling to look for plant pathways regulated by two *Albugo* species during infection. *Albugo* infection of Arabidopsis alters the profile of tryptophan-derived secondary metabolites, increasing camalexin accumulation and decreasing indol-3-ylmethylglucosinolate (I3M) levels. Interestingly, the camalexin accumulated in *Albugo*-infected tissue, though detectable in extracts, appears to be biologically unavailable for defense against the necrotrophic fungus *Botrytis cinerea. Albugo* also suppresses SATI, but lack of SA is not sufficient to allow colonization of Arabidopsis by *P. infestans*. Our results therefore suggest that *Albugo* affects many aspects of plant immunity, leading to the plant becoming susceptible to previously resisted pathogens, and that tryptophan-derived metabolites play a role in Arabidopsis NHR to *P. infestans*.

## Methods

### Biological material

Arabidopsis (*Arabidopsis thaliana*) plants were grown as previously described [12]. Seeds were sown on Scotts Levington F2 compost (Scotts, Ipswich, UK) and vernalized for one week at 5–6 °C. Seedlings were subsequently grown in a controlled environment room (CER) with a 10 h day and a 14 h night photoperiod and at a constant temperature of 22 °C for 2 weeks and then pricked out into “Arabidopsis mix” (600 L F2 compost, 100 L grit, 200 g Intercept insecticide) and returned to the CER. Arabidopsis plants were infected with *Albugo* when they were 4 or 5 weeks old. Arabidopsis lines used in this study are listed in Additional file 1.

*Brassica juncea* seeds were sown on Scotts Levington F2 compost (Scotts). Seedlings were subsequently grown in a CER with a 10 h day and a 14 h night photoperiod and at a constant temperature of 22 °C for 1 week and then pricked out into “Arabidopsis mix” and returned to the CER.

*Phytophthora infestans* isolate 88069td expresses a cytosolic tandem DsRed protein [44]. *P. infestans* isolate NL12226 was isolated by Geert Kessel (Wageningen University and Research, Wageningen) in 2012 from infected *Solanum tuberosum* cultivar Toluca in Valthermond, Flevoland, The Netherlands. Both isolates were cultured on rye sucrose agar [45] at 18 °C in the dark [46].

*Albugo* strains were propagated as follows: zoosporangia from plants inoculated 14 days earlier were suspended in cold water and incubated on ice for 30 min. The spore suspension was then sprayed on plants using a spray gun, and plants were incubated in a cold room (5 °C) in the dark overnight to promote *Albugo* spore germination. Infected plants were kept under 10-h light and 14-h dark cycles with a 21 °C day and 14 °C night temperature. *Albugo laibachii* strain Nc14 [47] was maintained on Col-gl *resistance to powdery mildew (RPW)8.1* and *RPW8.2* Arabidopsis [48]. *Albugo candida* (Ac) strains Ac2V [10], AcEx1 (this study), and AcNc2 [10] were maintained on *Brassica juncea* cultivar Burgonde, Col-0, and Ws-2 Arabidopsis ecotypes, respectively.

*Hyaloperonospora arabidopsidis* isolate Waco9 was inoculated as described previously [49, 50].

*Botrytis cinerea* was cultured and inoculated as described previously [51]. B05.10 is the wildtype strain. ΔBcatrB4 is a B05.10 derived gene-replacement mutant in BcatrB [52]. The BcatrB promoter–β-Glucuronidase (GUS) fusion strain BcatrBp803GUS-7 contains the 803 bp upstream of the BcatrB start codon fused in-frame to the *uidA* gene from *Escherichia coli* [53]. The OliCpromoter-GUS fusion strain OliCGUS shows constitutive expression of the *uidA* gene [53, 54].

### Gene expression analysis over *Albugo* infection time course

To harvest samples representing a time course of infection of *A. laibachii* and *A. candida* on Arabidopsis we have used a multi-parent recombinant inbred derived line, Multi-parent Advanced Generation Inter-Cross (MAGIC) 107 [55]. Arabidopsis ecotype Col-0 is resistant to AcNc2 and ecotype Ws-2 shows necrotic lesions, while MAGIC 107 shows significantly reduced trailing necrosis and exhibits a compatible interaction with AcNc2 and AlNc14. AcNc2 and AlNc14 were spray inoculated as described above. For mock treatment, plants were sprayed with cold water. Plants were incubated overnight in the dark at 5 °C. Arabidopsis leaf samples were collected immediately after the cold treatment (0 time point) and at 2, 4, 6, and 8 days post inoculation (dpi). Four independent biological replicates for each treatment and each time point were collected.

RNA extraction, EXpression Profiling through Randomly Sheared cDNA tag Sequencing (EXPRSS) library preparation for Illumina sequencing, and sequence read to gene mapping were performed as described previously [56]. Double stranded cDNA samples were sheared for library preparation using Covaris S220X (Covaris settings: intensity, 5; duty cycle, 20%; cycles/burst, 200; duration, 60 sec). The libraries were sequenced using Illumina Genome Analyzer II producing sequence reads of 76 nucleotides. The sequence data has been deposited in the National Center for Biotechnology Information’s Gene Expression Omnibus [57] and are available under series accession number GSE75016. Sequence reads to gene associations were carried out using the considerations and scripts previously published [56]. Mock samples were analyzed in pairwise manner with each *Albugo* species infection data, independently. Quality-filtered libraries of mock and AlNc14-infected samples were aligned to the combined genomes of The Arabidopsis Information Resource version 10 (TAIR10) [58] and AlNc14 version 1 [47]; similarly, mock and AcNc2-infected samples were aligned to combined genomes of TAIR10 and AcNc2 version 1 [10] using Bowtie version 0.12.8 [59]. Unaligned reads from previous steps were mapped to the combined cDNA reference sequences of the respective Arabidopsis (TAIR10) and *Albugo* strain (AlNc14 version1 and AcNc2 version1) combinations using Novoalign v2.08.03 [60]. Details of software parameters, genomes, and gene sequences used for the analysis are available online [61].

Uniquely aligned read counts were selected for differential expression analysis. For gene expression analysis, each *Albugo* (AlNc14 or AcNc2) infection time point data was compared against respective mock time point data resulting from pairwise analysis. Differential expression analysis was performed using the R statistical language [62] version 2.11.1 with the Bioconductor package [63] and edgeR version 1.6.15 [64] with the exact negative binomial test using tagwise dispersions. The Benjamini–Hochberg method [65] based false discovery rate (FDR) was applied and genes with FDR < 0.01 were selected as differentially expressed (Additional file 2).

For comparative analysis of benzo-(1,2,3)-thiadiazole-7-carbothioic acid (BTH) and JA responsive gene progression during *Albugo* infection, previously published microarray data of Arabidopsis treatment with BTH [66] and methyl jasmonate [67, 68] were used. Microarray data normalization and differential expression analysis was carried out as described previously [56]. Genes with FDR < 0.05 were selected for comparative gene expression analysis.

### Gene Ontology (GO) enrichment analysis

Lists of Arabidopsis genes that were up-regulated or down-regulated at each time point in infected plant tissue compared to the control were compiled (Additional file 3). Overlap between the AlNc14 and AcNc2 gene lists was determined using the Venn diagram available in the Public Research Centre for Health [69]. These lists were then used to perform Singular Enrichment Analysis with FDR = 0.05 using AgriGO v1.2 and default settings [70]. GO annotations are based on TAIR10.

### *P. infestans* infection assays

Sequential infection of plants with *Albugo* and then *P. infestans* were carried out with appropriate controls as previously described [12].

Assays with non-*Albugo*-infected Col-0 and mutant Arabidopsis were conducted by placing droplets of *P. infestans* spores on the abaxial side of detached leaves and incubating for up to 3 days at 100% relative humidity. After 36 hours, the droplets were gently removed using paper towel to prevent the growth of *P. infestans* in the water rather than the leaf.

### Visualizing and quantifying *P. infestans*

*P. infestans* 88069td colonization of Arabidopsis was visualized using a Leica M165FC microscope with DFC425 camera and EL6000 light source (Leica Microsystems, Milton Keynes, UK) and a DSR filter (excitation wavelength of 510–560 nm and emission wavelength of 590–650 nm). *P. infestans* growth is represented by red fluorescence. Leaves that were inoculated with *P. infestans* on the abaxial surface may show no fluorescence from the adaxial surface due to lack of pathogen colonization (e.g. Col-0 plants).

*P. infestans* colonization of Arabidopsis was quantified using qRT-PCR. Leaf discs (10 mm diameter) were punched out of Arabidopsis leaves inoculated with *P. infestans* and DNA extracted with DNeasy plant mini kit (Qiagen, Hilden, Germany). Four discs were used per replicate for water-sprayed plants, and three discs per replicate for *Albugo*-sprayed plants. DNA was diluted to 5 ng/μL and 5 μL used per qRT-PCR reaction. qRT-PCR was conducted as described below, using primers for At3g21215 and PiO8-3-3 (Additional file 4) to compare the amount of *P. infestans* DNA present.

*P. infestans* NL12226 sporulation on Col-0 and *cyp79b2/b3* Arabidopsis was quantified by infecting leaves from 4-week-old plants (as described above), then checking for the presence of *P. infestans* spores between 3 and 5 dpi by placing droplets of water on the leaf surface and examining them under a light microscope.

### qRT-PCR of plant genes

Plants were sprayed with *Albugo* or water, and subsequently inoculated with *P. infestans* as described above. Samples consisted of two Arabidopsis leaves and two samples were taken per experiment per time point, with the experiment being repeated three times.

Samples were homogenized using a TissueLyser II (Qiagen) and 3-mm tungsten carbide beads (Qiagen) under cold conditions. Total RNA was extracted using Tri-Reagent (Sigma-Aldrich), Direct-zol^TM^ RNA miniprep kit (Zymo Research, Irvine, CA), and on-column DNase treatment. Purity and integrity were checked using a Nanodrop 8000 (Thermo Scientific) and agarose gel. cDNA was synthesized from 1 μg RNA using Oligo dT_12–18_ primers (Life Technology, Paisley, UK) and Superscript III reverse transcriptase (Life Technology) according to the manufacturer’s instructions. cDNA from these reactions was diluted 1:20 with distilled water before qRT-PCR. Stable reference genes for normalization were selected as previously described [71]. Candidate reference genes were selected from previously identified superior reference genes [72] (Additional file 4). Analysis of eight candidates (*elongation factor 1 alpha*, *two A and related phosphatase-associated protein42-interacting protein of 41 kD* (*TIP41*), *U-BOX*, *glyceraldehyde-3-phosphate dehydrogenase C2*, *ACTIN2*, *PEROXIN4*, *monensin sensitivity1*, and *adaptor protein-2 MU-ADAPTIN*) using geNORM [73] and NormFinder [74] identified the optimal number of reference genes needed for normalization to be two, and the two most stable genes across the experimental conditions to be *TIP41* (At4g34270) and *elongation factor 1-alpha* (At5g60390). Primer sequences and annealing temperature used for qRT-PCR are described in Additional file 4.

### qRT-PCR assays

Each reaction consisted of 20 μL containing 5 μL of DNA or cDNA and 0.5 μM of each primer (Additional file 4) added to SYBR Green JumpStart Taq ReadyMix (Sigma-Aldrich) in a single well of a 96-well white ABgene PCR plate (Thermo Scientific). Reactions were run in a CFX96 Real-Time System with a C1000 Thermal Cycler (Bio-Rad). PCRs were carried out using the following thermocycle: 3 min at 95 °C, followed by 40 cycles of 30 s at 95 °C, 30 s at the relevant annealing temperature (Additional file 4), and 30 s at 72 °C, followed by melt curve analysis (65–95 °C at 0.5 °C increments, 5 s for each). Primer efficiencies were calculated using a dilution series of DNA or cDNA. To calculate the relative expression levels of target genes, mean cycle threshold values for each sample-primer pair combination were calculated from three replicate reaction wells. The cycle threshold values and primer efficiencies were then used to calculate normalized relative quantities (NRQs) for each gene using the EasyqpcR package [75] in R. NRQs were then log_2_ transformed [76] and statistical analyses performed as described below.

### Metabolite analysis

Plants were sprayed with *Albugo* or water, and subsequently inoculated with *P. infestans* or water as described above. Single leaves were collected 20 hours post *P. infestans*/control treatment for analysis of indolic glucosinolates and 48 hours post treatment for camalexin analysis.

Plants were sprayed with AlNc14 or water, and subsequently sprayed with *B. cinerea* or quarter-strength potato dextrose broth. Sets of three leaves were collected 26 hours post *B. cinerea*/control treatment for camalexin analysis. All samples were immediately flash frozen in liquid nitrogen and subsequently dry frozen.

Glucosinolates were analyzed as desulfo glucosinolates through a modified version of a previously described method [77]. Leaf material was lyophilized and homogenized in 85% methanol containing 0.02 mM *para*-hydroxybenzyl glucosinolate as internal standard. Samples were centrifuged at 13,000 *g* for 10 min and the supernatant was transferred to a 96-well filter plate (Millipore) loaded with 45 mg diethylaminoethyl sephadex^TM^ A-25 column material (GE Healthcare Biosciences), which had been equilibrated for 4 hours in 300 μL water before samples were applied. Glucosinolates were bound to the column material while samples were sucked through the filter plate by applying a brief vacuum. Afterwards, columns were washed with 2 × 100 μL 70% methanol and 2 × 100 μL water, respectively. Then, 20 μL sulfatase (SIGMA E.C. 3.1.6.) solution (2 mg mL^–1^) was added to the columns and allowed to incubate at room temperature overnight; 100 μL water were applied to the columns and a short spin eluted the desulfo-glucosinolates into a 96-well format plate. The samples were analyzed on a Shimadzu high performance liquid chromatography (HPLC)-DAD system and separated on a Zorbax SB-AQ column (4.6 mm × 25 cm, 5 μm particle size) at a flow rate of 1 mL min^–1^. Compounds were detected at 229 nm using a diode array UV and separated utilizing eluents (A: H_2_O, B: 100% acetonitrile) using the following program: 5 min gradient from 1.5% to 7% eluent B; 5 min gradient from 7% to 25% eluent B; 4 min gradient from 25% to 80% eluent B; 3 min at 80% eluent B; 2 min gradient from 80% eluent B to 35% eluent B; 2 min gradient from 35% to 1.5% eluent B; a final 3 min at 1.5% eluent B. Response factors for absorbance at 229 nm were used to quantify the desulfo-glucosinolates [78–80].

Leaf samples for camalexin analysis were disrupted in methanol using a Retsch Mixer Mill 303 (Retsch, Haan, Germany). Samples were spun down and the supernatant collected, and the process was repeated with the pellet tissue. Supernatants were filtered through a 0.22-μm filter (Millipore). Samples were quantified using synthetic camalexin as an external standard. The peak at 5.17 min was identified as camalexin by comparison with authentic standard with respect to retention time and UV spectrum (photodiode array detector 168, Beckman Instruments, Fullerton, CA) and quantified by using a Shimadzu F-10AXL fluorescence detector (318 nm excitation and 370 nm emission) and by UV absorption at 318 nm.

#### Botrytis cinerea

Inoculation of *Arabidopsis* with *B. cinerea* was performed as described previously [81], with minor modifications. For disease assays, plants sprayed with AlNc14 or water 12 days previously were pairwise-inoculated with the different isolates using 5 μL droplets of 2.5 × 10^5^ spores per mL in quarter-strength potato dextrose broth. Six leaves per plant and at least eight plants per experiment were used. Lesion diameters were measured at 3 dpi.

For determination of GUS activity in OliCGUS and BcatrBp803GUS-7 water- or AlNc14-sprayed leaves were inoculated by pairwise droplet inoculation of three droplets of each strain on either side of the leaf or sprayed as a whole plant till near run-off. For visual examination of the droplets inoculated leaves were detached at 48 hours post inoculation (hpi) and vacuum-infiltrated three times for 2 mins in X-Gluc staining buffer (50 mM sodium phosphate buffer pH 7.0, 10 mM ethylenediaminetetraacetic acid (EDTA), 0.5 mM K_3_Fe(CN)_6_, 0.5 mM K_4_Fe(CN)_6_, 0.5% w/v Triton X-100 and 0.5 mg mL^−1^ X-Gluc cyclohexylammonium salt) [51, 82]. Leaves were incubated for 20 h at 37 °C, destained in four changes of ethanol, and the intensity of blue staining at each inoculation site was estimated on a scale from 0 to 3. The score of all droplets per leaf was averaged and expressed as percentage of the maximum per leaf and data presented are averages of three experiments with at least five leaves per pairwise comparison. For determination of GUS activity in sprayed leaves, three leaves were collected 48 hpi, blotted dry on tissue paper, weighed and frozen in 2-mL tubes. Leaves in each tube were pulverised in a genogrinder 2010 [83] with two 3-mm stainless steel balls for 1 min at 1250 strokes per minute in blocks cooled with dry-ice. Enzymes were extracted with 25 mM sodium phosphate buffer pH 7.0 with 0.1% Triton and GUS activity determined as the conversion of 4-methylumbelliferyl-β-D-glucuronide (Sigma) by GUS to its fluorescent degradation product on a Varioskan Flash multiplate reader (Thermo Scientific) adapted from Jefferson et al. [84]. The remaining pellet was used for total DNA extraction and qRT-PCR determination of *B. cinerea* levels in each sample according to Gachon et al. [85] (Additional file 4). GUS expression was normalized against the *B. cinerea* weight portion of each sample.

### Microscopy of *PR1*::GUS leaves

GUS activity in leaves of *pathogenesis-related 1 (PR1)*::GUS plants was assayed histochemically with 5-bromo-6-chloro-3-indolyl b-D-glucuronide cyclohexylammonium salt (1 mg mL^–1^) (Magenta b-D-GlcA CHX, Carbosynth Limited, Compton, UK) in a buffer containing 100 mM sodium phosphate pH 7.0, 0.5 mM potassium ferrocyanide (Sigma-Aldrich, St Louis, USA), 0.5 mM potassium ferricyanide (Sigma-Aldrich), 10 mM EDTA (Thermo Scientific, Loughborough, UK), and 0.1% Triton (Sigma-Aldrich). Arabidopsis leaves were vacuum-infiltrated with staining solution and incubated overnight at 37 °C in the dark. Leaves were then boiled in lactophenol containing 0.17 mg mL^–1^ trypan blue (Sigma-Aldrich) for 1 min and destained by incubation in 2.5 g mL^–1^ chloral hydrate (Sigma-Aldrich). Staining of whole leaves was visualized using a Leica M165FC microscope with DFC425 camera and EL6000 light source (Leica Microsystems). The percentage of the leaf stained with magenta-GlcA was determined by measuring the leaf area and the stained area using ImageJ [86].

### Statistical analyses

Statistical analyses were conducted using R 3.2.2 [62] within RStudio 0.99.483 [87] (data are available in Additional files relating to each figure; please see below). Technical replicates consist of readings from the same condition in the same experiment, whereas biological replicates consist of independent experiments with batches of plants sown on different days. Data were analyzed using the following pipeline: data were assessed for their suitability to be analyzed using parametric tests by testing for the normal distribution of the residuals (D’Agostino–Pearson and Shapiro–Wilk tests) and visualizing residuals with Q-Q plots. The assumption of equal variances between the conditions was tested using the Bartlett test for data with normally distributed residuals and the Fligner test for data with non-normally distributed residuals. If the data were suitable for conducting parametric tests, then Welch’s two sample t-test or analysis of variance (ANOVA) were used as appropriate. Percentage data in Additional file 5 were transformed in order to meet the assumptions of parametric tests. The percentage of leaf stained was first arcsine square root transformed (arcsine(square root(percentage/100))), and then subsequently log_10_ transformed (log_10_(transformed data point + 1)). If the data were not suitable for parametric tests, then the appropriate non-parametric test (Wilcoxon rank sum test, Kruskal–Wallis rank sum test) was used if possible. Data that did not meet the assumptions for parametric tests but had more than one set of treatments were analyzed within a generalized linear model (GLM) using a Poisson distribution, or a quasi-Poisson distribution if the data were over dispersed. Multiple comparisons were corrected for using Tukey’s honest significant difference (HSD) where appropriate, and otherwise Bonferroni correction.

## Results

### Two *Albugo* species compromise plant immunity and enables sporulation of *Phytophthora infestans*

**Fig. 1:**
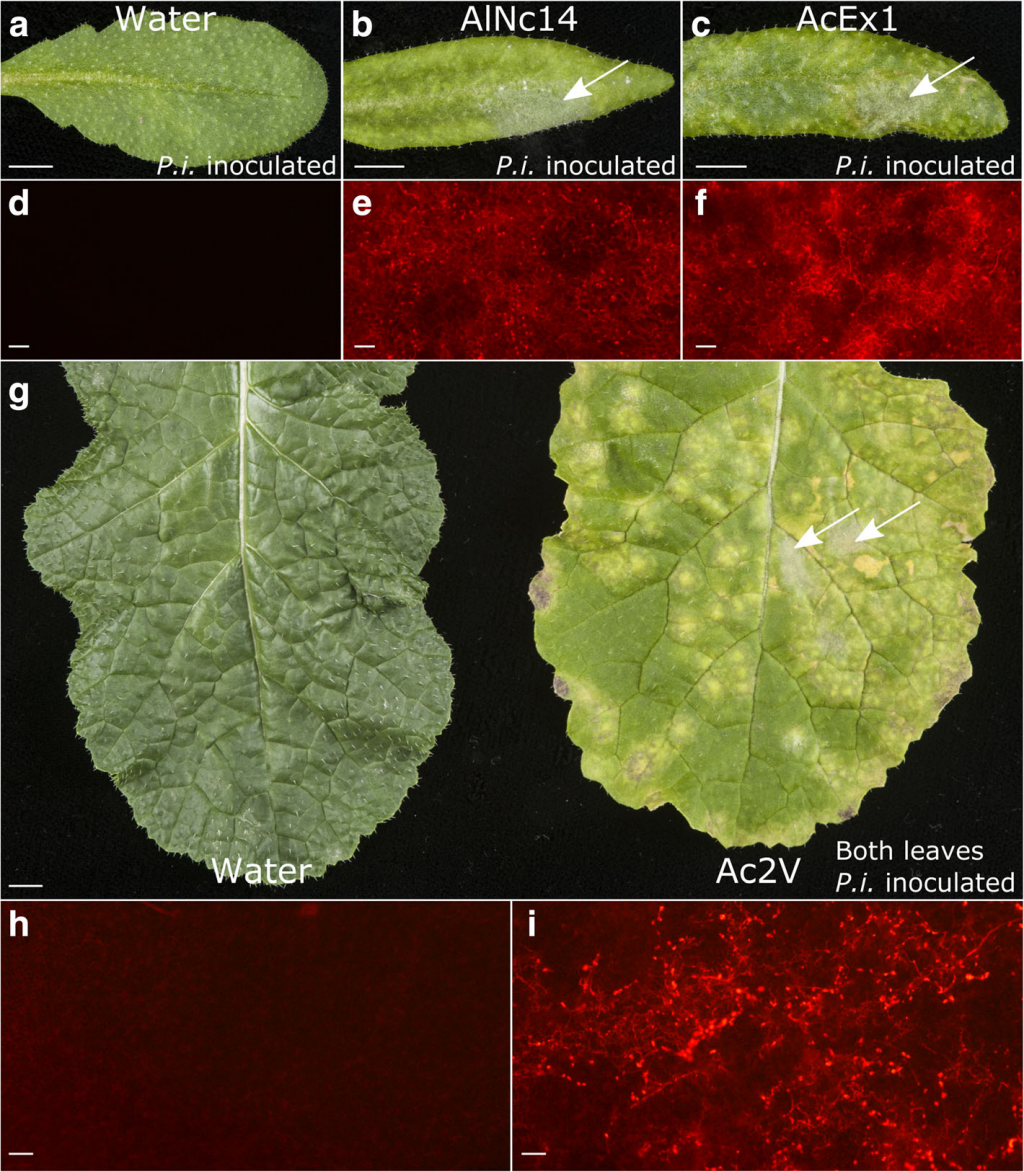
Two *Albugo* species compromise plant immunity and enable sporulation of *Phytophthora infestans.* **a–f** *Albugo* species compromise Arabidopsis immunity to *P. infestans.* Water-sprayed (**a, d**), *Albugo laibachii* Nc14-sprayed (**b, e**), and *Albugo candida* AcEx1-sprayed (**c, f**) Col-0 leaves (13 days post inoculation (dpi)) were drop inoculated with 100 μL of 5 × 10^4^ spores per mL *P. infestans* 88069td. **a–c** Photographs taken 3 dpi with *P. infestans.* Scale bar: 5 mm. Arrows denote *P. infestans* sporulation. **d–f** Fluorescence microscopy of the adaxial surface of the leaf. Red fluorescence denotes *P. infestans* growth. Scale bar: 200 μm. Results shown are representative of three independent experiments. **g–i** *A. candida* compromises *Brassica juncea* immunity to *P. infestans.* **g** Water-sprayed (left) and *A. candida* Ac2V-infected (right) *B. juncea* leaves (12 dpi) were drop inoculated with several 250 μL drops of 4 × 10^4^ spores per mL *P. infestans* 88069td. Photographs were taken 3 dpi with *P. infestans.* Scale bar: 5 mm. Arrows denote *P. infestans* sporulation. **h, i** Fluorescence microscopy of the adaxial surface of water-sprayed (**h**) and Ac2V-infected (**i**) leaves. Red fluorescence denotes *P. infestans* growth. Scale bar: 200 μm. Results shown are representative of three independent experiments

We recently reported that *A. laibachii* Nc14 (AlNc14) [47] suppresses Arabidopsis NHR to *P. infestans* ([12], Fig. 1a, b, d and e). As immunosuppression was also demonstrated for the related species *A. candida* [10, 11], we investigated whether *A. candida* infection of Arabidopsis and *Brassica juncea* compromises NHR to *P. infestans. A. candida* isolate Exeter 1 (AcEx1), which is adapted to many Arabidopsis ecotypes including Col-0, suppressed NHR in Arabidopsis to *P. infestans* (Fig. 1c and f). *A. candida* isolate 2V (Ac2V) is adapted to *B. juncea* but not Arabidopsis ecotypes [10], and also suppresses plant NHR to *P. infestans* on *B. juncea* (Fig. 1g–i). *P. infestans* sporulates in both AcEx1- and Ac2V- infected leaves (Fig. 1c, f, g and i). To test if the NHR suppression was imposed by other biotrophic oomycetes that infect Arabidopsis, we inoculated *Hyaloperonospora arabidopsidis* (*Hpa*)-infected Arabidopsis with *P. infestans*. We saw no *P. infestans* colonization of Arabidopsis infected with the compatible *Hpa* isolate Waco9 (Additional file 6). Together, these data suggest that suppression of NHR to *P. infestans* is imposed after infection by *Albugo* species but not by other biotrophic oomycete pathogens of Arabidopsis.

### *Albugo*-infection upregulates plant tryptophan metabolism

**Fig. 2:**
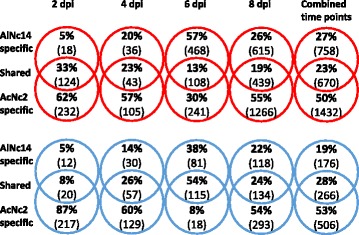
Genes differentially expressed in expression profiling experiment. The number of differentially expressed genes in MAGIC 107 Arabidopsis infected with AlNc14 or AcNc2 was calculated over an 8-day time course. The data are the average of four experiments. The Venn diagrams show the percentage of genes (with number of genes in brackets) that were up-regulated (red rings) or down-regulated (blue rings) at that time point and whether they were either unique to infection with one pathogen species, or were shared between the two pathogen species. Combined time points show genes that were up-regulated at one or more time points and not subsequently down-regulated (and vice versa)

To understand the effect of *Albugo* infection on plant gene expression over a time course of infection we used EXPRSS, a sensitive, reliable, and high-throughput tag-based expression profiling method [56]. We wished to compare the Arabidopsis gene expression responses to infection with two *Albugo* species, AlNc14 and *A. candida* isolate Nc2 (AcNc2). While AlNc14 is compatible with many Arabidopsis ecotypes, Col-0 is resistant to AcNc2 and Ws-2 shows necrotic lesions upon AcNc2 infection. Arabidopsis MAGIC line 107 [55] was chosen after screening multiple MAGIC lines because it shows the most compatible interaction (significantly reduced trailing necrosis) with AcNc2, and also showed compatibility with AlNc14. We hypothesized that both species of *Albugo* suppress NHR to *P. infestans* by similar mechanisms. We treated MAGIC line 107 [55] with AlNc14, AcNc2 [10], or water as a control, and then took leaf samples for RNA extraction at 0, 2, 4, 6, and 8 dpi. EXPRSS libraries were prepared from the extracted RNA and sequenced using Illumina sequencing. The sequences were mapped to genes, and differential expression analysis conducted. There was some overlap in the Arabidopsis genes differentially regulated by both pathogen species, with around 25% of the total up-regulated and down-regulated genes across the time course shared by the two pathogen species (Fig. 2). To identify which plant pathways were altered by *Albugo*, we conducted GO enrichment analysis using AgriGo [70] on lists of differently expressed genes (Additional files 7 and 8), focusing on specific lower level terms within biological processes. Few plant pathways were up-regulated at early time points in both infections (Table 1). At later time points, pathways associated with plant defense, such as SA and JA, were up-regulated. The only enriched down-regulated plant processes shared by infection with either pathogen were photosynthesis and RNA elongation. We focused on the up-regulation of the tryptophan-derived secondary metabolites, which include camalexin and indole-derived compounds, as these pathways were enriched in genes up-regulated by AlNc14 and AcNc2 infection (Table 1; 8 dpi and Combined time points), and they have been shown to play a role in Arabidopsis immunity to other *Phytophthora* species [31, 32].

**Table 1:** Gene ontology (GO) terms enriched in Arabidopsis genes differentially expressed by both pathogen infections

Category	2 dpi	4 dpi	6 dpi	8 dpi	Combined time points
Up-regulated vs. control (0 dpi)	• Golgi apparatus	• rRNA modification	• Jasmonic acid-mediated signaling pathway • MAPKKK cascade • Negative regulation of programmed cell death • Salicylic acid-mediated signaling pathway • Systemic acquired resistance	• Indole derivative biosynthetic processes • Jasmonic acid-mediated signaling pathway • MAPKKK cascade • Negative regulation of programmed cell death • Response to hormone stimulus • Salicylic acid-mediated signaling pathway • Systemic acquired resistance • Tryptophan metabolic processes	• Camalexin biosynthetic processes • Indole-derived metabolic processes • Jasmonic acid-mediated signaling pathway • MAPKKK cascade • Negative regulation of defense response • Negative regulation of programmed cell death • Response to hormone stimulus • Salicylic acid-mediated signaling pathway • Systemic acquired resistance • Tryptophan metabolic processes
Down-regulated vs. control (0 dpi)	• Photosynthesis • RNA elongation	• Photosynthesis • RNA elongation	• Photosynthesis • RNA elongation	• Photosynthesis • RNA elongation	• Photosynthesis • RNA elongation

### *Albugo* infection changes the proportions of camalexin and indolic glucosinolates

**Fig. 3:**
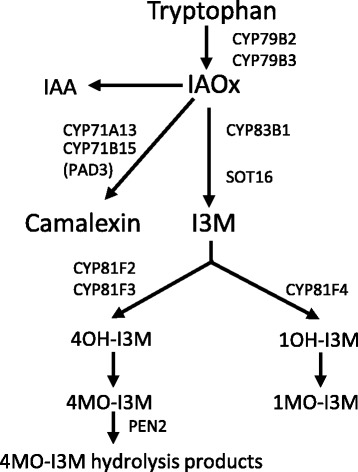
The tryptophan-derived metabolite pathway. Simplified schematic of the tryptophan-derived metabolite pathway, adapted from Buxdorf et al. [113] and Frerigmann et al. [114]

**Fig. 4:**
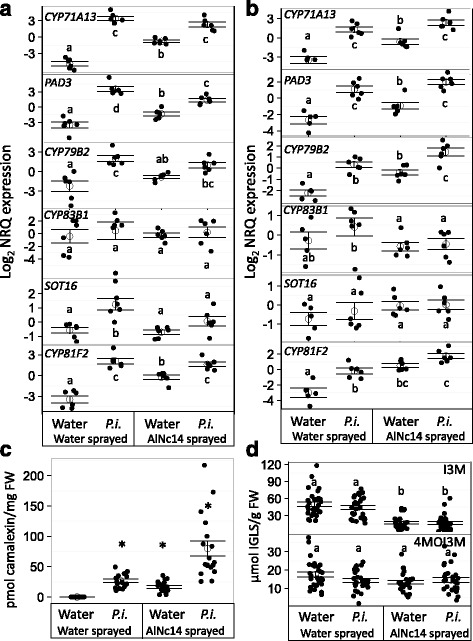
*Albugo* infection changes the proportions of camalexin and indolic glucosinolates but does not eliminate them. **a** and **b** *Albugo* infection changes expression of selected genes in the tryptophan-derived metabolite pathway upon *P. infestans* infection. Open circles and bars denote the mean ± SE of target gene expression (log_2_ transformed normalized relative quantities) in water-sprayed or AlNc14-infected tissue after water or *P. infestans* (100 μL of 1.25 × 10^5^ spores per mL) inoculation. **a** 10 days post inoculation (dpi) with water or AlNc14, 6 hours post inoculation (hpi) with water or *P. infestans.* **b** 12 dpi with water or AlNc14, 48 hpi with water or *P. infestans*. Data are three independent biological replicates with two technical replicates each. Closed, black circles denote individual data points. Different letters indicate significant differences (*P* < 0.05) (Two-way ANOVA with Tukey’s honest significance difference). **c** *Albugo* and *P. infestans* infection triggers camalexin accumulation. High-performance liquid chromatography (HPLC) analysis of water-sprayed or AlNc14-infected Col-0 tissue (12 dpi), 48 hours post water or *P. infestans* inoculation (100 μL of 2.75 × 10^5^ spores per mL). Open circles and bars denote the mean camalexin content per mg fresh weight ± SE of three independent biological replicates with six technical replicates each. Closed, black circles denote individual data points. Asterisks indicate significant differences from mock-treated plants (12 dpi water, 48 hpi water). Generalized linear model (GLM) with *P < 0.001. **d** *Albugo* infection decreases I3M levels but does not affect 4MO-I3M levels. HPLC analysis of mock or AlNc14-infected Col-0 tissue (12 dpi), 20 hpi mock or *P. infestans* (100 μL of 3 × 10^5^ spores per mL). Open circles and bars denote the mean indolic glucosinolate content per g of fresh weight ± SE of five independent biological replicates with six technical replicates each. Closed, black circles denote individual data points. GLM with different letters indicating significant differences (*P* < 0.001)

To explore whether tryptophan-derived secondary metabolites are involved in Arabidopsis responses to *P. infestans* and how *Albugo* infection may alter their accumulation, we measured Arabidopsis transcriptional responses and metabolite accumulation in water-sprayed and *Albugo*-infected plants in response to *P. infestans*. We selected genes that were at the start of the pathway (*cytochrome P450* (*CYP)79B2*), on the camalexin branch (*CYP71A13* and *phytoalexin deficient3* (*PAD3*)), on the core indolic glucosinolate pathway (*CYP83B1* and *sulfotransferase16* (*SOT16*)), and involved in indolic glucosinolate modification (*CYP81F2*) (Fig. 3). At 6 hours (Fig. 4a, Additional files 9 and 10), *Albugo* infection alone up-regulated *CYP71A13*, *PAD3*, and *CYP81F2. P. infestans* infection alone up-regulated all of the genes except *CYP83B1. SOT16* expression induced by *P. infestans* was suppressed in the presence of *Albugo*. At 48 hours (Fig. 4b, Additional files 10 and 11), *Albugo* infection alone up-regulated the same genes as at 6 hours plus *CYP79B2. P. infestans* infection alone up-regulated the same genes as at 6 hours, with the exception of *SOT16. Albugo* and *P. infestans* infection together led to increased expression of *CYP79B2* and *CYP81F2*, and decreased expression of *CYP83B1* compared to *P. infestans* infection alone. These data support the inference of the expression profiling and GO enrichment analysis that genes involved in tryptophan-derived secondary metabolite processes are up-regulated in *Albugo*-infected tissue. They also show that these genes respond to *P. infestans* infection.

We measured camalexin and indolic glucosinolate (I3M and 4MO-I3M) levels in leaves with the same experimental design as above. *Albugo*-treatment (t = –6.037, *P* < 0.001, GLM) and *P. infestans* inoculation (t = –7.340, *P <* 0.001) led to significant accumulation of camalexin (Fig. 4c, Additional file 10). *Albugo*-infected tissue accumulates significantly less I3M (t = 5.884, *P* < 0.001, GLM) but *P. infestans* inoculation has no effect (t = 0.037, *P* = 0.971) (Fig. 4d, Additional file 10). Neither of the treatments change the accumulation of 4MO-I3M (*Albugo*: t = –0.123, *P* = 0.90, *P. infestans*: t = –0.762, *P* = 0.45, GLM) (Fig. 4d, Additional file 10). 4MO-I3M accumulates in the *pen2-1* mutant upon challenge with flg22 or non-host pathogens due to reduced hydrolysis [18, 88]. However, we found similar results to Col-0 when we repeated the experiment in the *pen2-1* mutant (Additional files 12 and 13). In conclusion, *P. infestans* infection of Arabidopsis elicits transcriptional responses in the camalexin and indolic glucosinolate metabolic pathways, and the accumulation of camalexin. *Albugo*-infection appears to alter tryptophan-derived secondary metabolite levels leading to increased accumulation of camalexin and decreased accumulation of I3M.

### Indole glucosinolate-deficient, but not aliphatic glucosinolate-deficient mutants, show reduced resistance to *P. infestans*

**Fig. 5:**
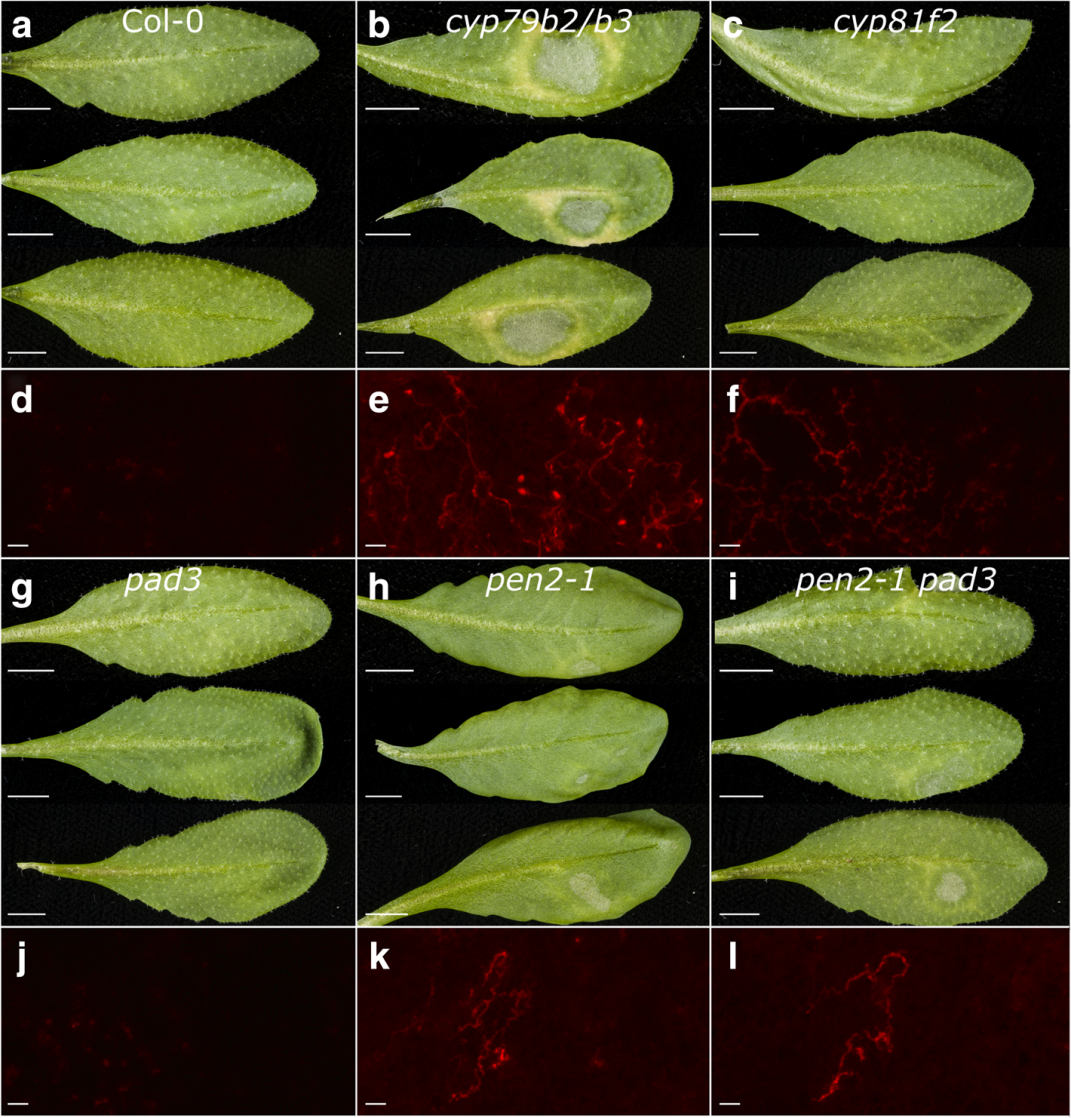
*P. infestans* successfully colonizes *cyp79b2*/*b3. P. infestans* colonization of mutants in the tryptophan-derived metabolite pathway. **a–c**, **g–i** Leaves were inoculated with 100 μL of 1 × 10^5^ spores per mL *P. infestans* 88069td and photographed at 3 dpi. Scale bar: 5 mm. Leaves from three independent experiments are shown. **d–f**, **j–l** Adaxial surface of the leaves was examined using fluorescence microscopy at 3 dpi. Scale bar: 100 μm. Three independent experiments were conducted, microscopy from one of the experiments is shown

**Fig. 6:**
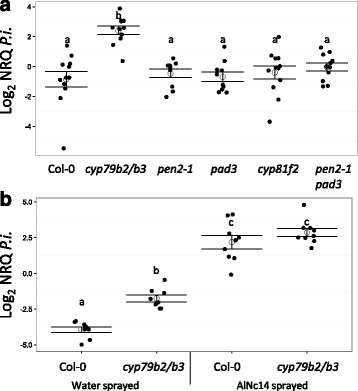
*P. infestans* shows increased biomass on *cyp79b2/b3* compared to Col-0 in the absence of *Albugo*, but not in its presence. **a** *P. infestans* biomass on mutants in the tryptophan-derived metabolite pathway. Leaves were inoculated with 100 μL of 1 × 10^5^ spores per mL *P. infestans* 88069td. DNA was extracted at 3 dpi and the proportion of *P. infestans* DNA to plant DNA determined using qRT-PCR. Open circles and bars denote the mean ± SE of *P. infestans* DNA (log_2_ transformed normalized relative quantities (NRQs)) in Arabidopsis tissue from four independent biological replicates with three technical replicates per biological replicate. Closed, black circles denote the individual data points. Different letters indicate significant differences (*P* < 0.01) (Kruskal–Wallis rank sum test with Dunn multiple comparisons test and Bonferroni correction). **b** Higher *P. infestans* biomass on AlNc14*-*infected Arabidopsis than on *cyp79b2/b3*. Leaves were inoculated with 100 μL of 1 × 10^5^ spores per mL *P. infestans* 88069td. DNA was extracted at 3 dpi and the proportion of *P. infestans* DNA to plant DNA determined using qRT-PCR. Open circles and bars denote the mean ± SE of *P. infestans* DNA (log_2_ transformed NRQs) in Arabidopsis tissue from three independent biological replicates with three technical replicates per biological replicate. Closed, black circles denote the individual data points. Different letters indicate significant differences (*P* < 0.01) (Two-way ANOVA with Tukey’s honest significance difference test)

To further investigate the role of tryptophan-derived secondary metabolites in NHR to *P. infestans* we selected mutants deficient in different parts of the pathway. We tested NHR to *P. infestans* in mutants deficient in indolic glucosinolates and camalexin (*cyp79b2/b3*), deficient in camalexin (*pad3*), reduced in 4MO-I3M (*cyp81f2*), deficient in *PEN2-*dependent hydrolysis of 4MO-I3M (*pen2-1*), and deficient in *PEN2-*dependent hydrolysis of 4MO-I3M and camalexin (*pen2-1 pad3*) (Fig. 3). *cyp79b2/b3*, *pen2-1*, and *pen2-1 pad3* showed cell death in response to *P. infestans* inoculation, with the strongest phenotype observed with *cyp79b2/b3* (Fig. 5b, h and i). These observations were complemented by fluorescence microscopy, which revealed that *cyp79b2/b3*, *cyp81f2*, *pen2-1*, and *pen2-1 pad3* allowed *P. infestans* growth within the leaf that was visible from the adaxial surface (Fig. 5e, f, k and l). *P. infestans* was observed to form haustoria (Additional file 14) and occasionally sporulate (between 0 and 8.9% of leaves; Additional file 15, Fig. 5e) during infection of *cyp79b2/b3* tissue. We quantified the relative amount of *P. infestans* biomass on each mutant compared to Col-0 using qRT-PCR. In agreement with microscopy, *P. infestans* biomass was significantly higher on *cyp79b2/b3* than Col-0 or the other mutants (*P* < 0.05, Fig. 6a, Additional file 16). We also tested the susceptibility to *P. infestans* of an Arabidopsis line that overproduces brassinosteroid and was reported to have a similar I3M and 4MO-I3M profile to *Albugo*-infected plants (*35S:DWF4* (*DWARF4*) [89]). *35S:DWF4* was not compromised in NHR to *P. infestans* (Additional files 16). Surprisingly, *P. infestans* grew less well on *35S:DWF4* plants infected with AlNc14 than on Col-0 plants infected with AlNc14 (Additional file 13 and 16).

Having identified *cyp79b2/b3* as compromised in NHR to *P. infestans* we then investigated whether *cyp79b2/b3* acts in the same pathway as *Albugo* in Arabidopsis NHR to *P. infestans.* We infected water- and AlNc14-sprayed Col-0 and *cyp79b2/b3* Arabidopsis with *P. infestans* and quantified *P. infestans* biomass with qRT-PCR. *Albugo-*infected Col-0 and *Albugo*-infected *cyp79b2/b3* had the same degree of *P. infestans* colonization, which was significantly higher than water-sprayed *cyp79b2/b3*, which in turn was significantly higher than water-sprayed Col-0 (pre-treatment: F_(1, 30)_ = 270.1, *P* < 0.001, genotype: F_(1, 30)_ = 18.36, *P* < 0.001, interaction: F_(1, 30)_ = 5.347, *P* = 0.028; two-way ANOVA with Tukey’s HSD) (Fig. 6b, Additional file 17). *Albugo*-infected Col-0 and *Albugo*-infected *cyp79b2/b3* were more susceptible to *P. infestans* than water-sprayed *cyp79b2/b3*, suggesting that deficiency in tryptophan-derived metabolites does not solely explain *Albugo-*immunosuppression.

To further investigate the role of glucosinolates in *P. infestans* NHR we tested whether aliphatic glucosinolates, which are not derived from tryptophan, play a role. We infected the *myb28/29* double mutant, which does not accumulate aliphatic glucosinolates [90], with *P. infestans. myb28/29* did not allow colonization by *P. infestans* (Additional file 18). We also tested *thioglucoside glucohydrolase* (*tgg)1/tgg2*, a mutant in two myrosinases expressed in aerial tissue [91]. *P. infestans* did not colonize *tgg1/tgg2* (Additional file 18). We therefore conclude that aliphatic glucosinolates play a minimal role in *P. infestans* NHR. In summary, *Albugo*-suppression of *P. infestans* NHR involves tryptophan-derived secondary metabolites. However, given the increase in *P. infestans* colonization between water-sprayed and Albugo-infected *cyp79b2/b3*, we conclude that additional changes are imposed by *Albugo* infection, which promotes *P. infestans* susceptibility.

### *Albugo*-induced camalexin is biologically unavailable to *Botrytis cinerea*

**Fig. 7:**
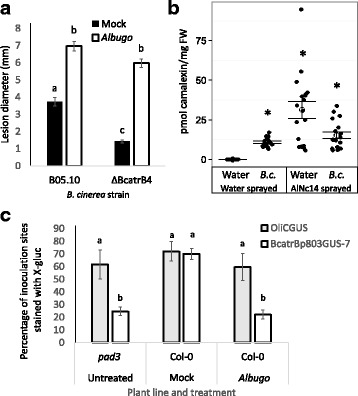
*Albugo*-induced camalexin is biologically unavailable to *Botrytis cinerea.* **a** *B. cinerea* gives increased disease symptoms on *Albugo*-infected tissue. Leaves of water-sprayed or AlNc14-infected Col-0 Arabidopsis (11 dpi) were inoculated with 2.5 × 10^5^ spores per mL of *B. cinerea* B05.10 or camalexin sensitive ΔBcatrB4 mutant, and lesion diameters were measured at 2 dpi. Bars represent mean lesion diameter ± SE of three independent biological replicates with between 7 and 11 technical replicates per biological replicate (n = 28). Different letters indicate significant differences between treatments at *P* < 0.01 (Two-way ANOVA with Tukey’s honest significance difference). **b** Camalexin accumulates in plants infected by *Albugo* and *B. cinerea*, either alone or together. High-performance liquid chromatography (HPLC) analysis of mock or AlNc14-infected Col-0 tissue (12 dpi), 26 hours post mock or *B. cinerea* B05.10 inoculation by spraying (2.5 × 10^5^ spores per mL). Open circles and bars denote mean camalexin content per mg of fresh weight ± SE of three independent biological replicates with six technical replicates per biological replicate. Closed, black circles denote individual data points. Asterisks indicate significant differences from mock treated plants (12 days post water spraying, 26 hours post inoculation) at *P* < 0.001 (Generalized linear model (GLM)). **c** *B. cinerea* detects less available camalexin in *Albugo*-infected tissue. Leaves of mock or AlNc14-infected Arabidopsis (11 dpi) were drop inoculated with 2.5 × 10^5^ spores per mL of *B. cinerea* strains OliCGUS (constitutive GUS expression) or BcatBp803GUS-7 (camalexin inducible GUS expression). Leaves were stained with X-gluc at 2 dpi and the percentage of infection sites showing staining determined. Bars represent mean ± SE three independent biological replicates with between two and four technical replicates per biological replicate (bars left to right n = 5, 8, 7, 10, 5, 8). Different letters indicate significant differences *P* < 0.05 (Three-way ANOVA, Tukey’s honest significant difference test)

*Albugo*-infected plants accumulated camalexin (Fig. 4c), which is toxic to necrotrophic fungi including *Botrytis cinerea* [51, 92, 93]. We therefore tested whether *Albugo*-infected plants had altered susceptibility to *B. cinerea* by measuring the growth of *B. cinerea* wild type strain B05.10 and mutant ΔBcatrB4 (lacking a detoxifying ABC exporter) on water-sprayed and *Albugo-*infected plants. ΔBcatrB4 was more susceptible to camalexin and had reduced virulence on Col-0 but not on the camalexin-deficient mutant *pad3* [51]. We found that *B. cinerea* B05.10 infection of *Albugo*-infected plants resulted in lesions almost twice as big as on water-sprayed plants (Fig. 7a). The camalexin sensitive ΔBcatrB4 mutant grew significantly less well on water-sprayed plants but produced lesions of a similar size to wild type B05.10 on *Albugo*-infected plants (Pre-treatment: F_(1, 104)_ = 305.9, *P* < 0.001, strain: F_(1, 104)_ = 56.31, *P* < 0.001, interaction: F_(1, 104)_ = 8.713, *P* < 0.01; two-way ANOVA with Tukey’s HSD) (Fig. 7a, Additional file 19). Next, we quantified the accumulation of camalexin in response to *B. cinerea* B05.10 and AlNc14. *Albugo* treatment (z = –3.409, *P* < 0.001, GLM) and *B. cinerea* inoculation (z = 9.784, *P <* 0.001) led to significant accumulation of camalexin, although the interaction between the two treatments was not significant (z = –0.025, *P* = 0.980) (Fig. 7b, Additional file 19). Therefore, the increased susceptibility of *Albugo*-infected plants to *B. cinerea* is not due to an overall lack of camalexin accumulation. On the contrary, it suggests that, after *Albugo* infection, camalexin levels no longer restrict *B. cinerea* proliferation, as lesion sizes are similar in the presence or absence of the detoxifying transporter BcatrB. To assess whether *B. cinerea* encounters the camalexin present in the plant tissue we used a BcatrB promoter–GUS fusion strain of *B. cinerea* (BcatrBp803GUS-7). BcatrBp803GUS-7 has low basal expression and is inducible by camalexin [51, 54]. As a control for GUS staining we used the OliCpromoter-GUS fusion *B. cinerea* strain OliCGUS, which shows constitutive expression of the reporter [53, 54]. We also used *pad3* to assess the background expression of BcatrBp803GUS-7 in the absence of camalexin. The two *B. cinerea* GUS-strains showed similar staining on water-sprayed Col-0 plants but on *Albugo*-infected Col-0 plants the GUS expression in BcatrBp803GUS-7 was reduced significantly to levels comparable to when the same strain infected *pad3* plants (*P* = 0.002) (Pre-treatment: F_(1, 37)_ = 13.449, *P* < 0.001, strain: F_(1, 37)_ = 19.39, *P* < 0.001, genotype: F_(1, 37)_ = 26.559, *P* < 0.00, interaction between strain and genotype: F_(1, 37)_ = 13.449, *P* < 0.01; three-way ANOVA with Tukey’s HSD) (Fig. 7c, Additional file 19 and 20). The reduction in GUS production by BcatrBp803GUS-7 on *Albugo-*infected plants was confirmed by quantifying GUS enzymatic activity using 4-methylumbelliferyl-beta-D-glucuronide (Additional files 13 and 21). These results suggest that, in *Albugo*-infected plants, *B. cinerea* is exposed to lower camalexin levels than might be expected based on camalexin level measurements in whole leaves.

### SA regulated genes during *Albugo* infection

**Fig. 8:**
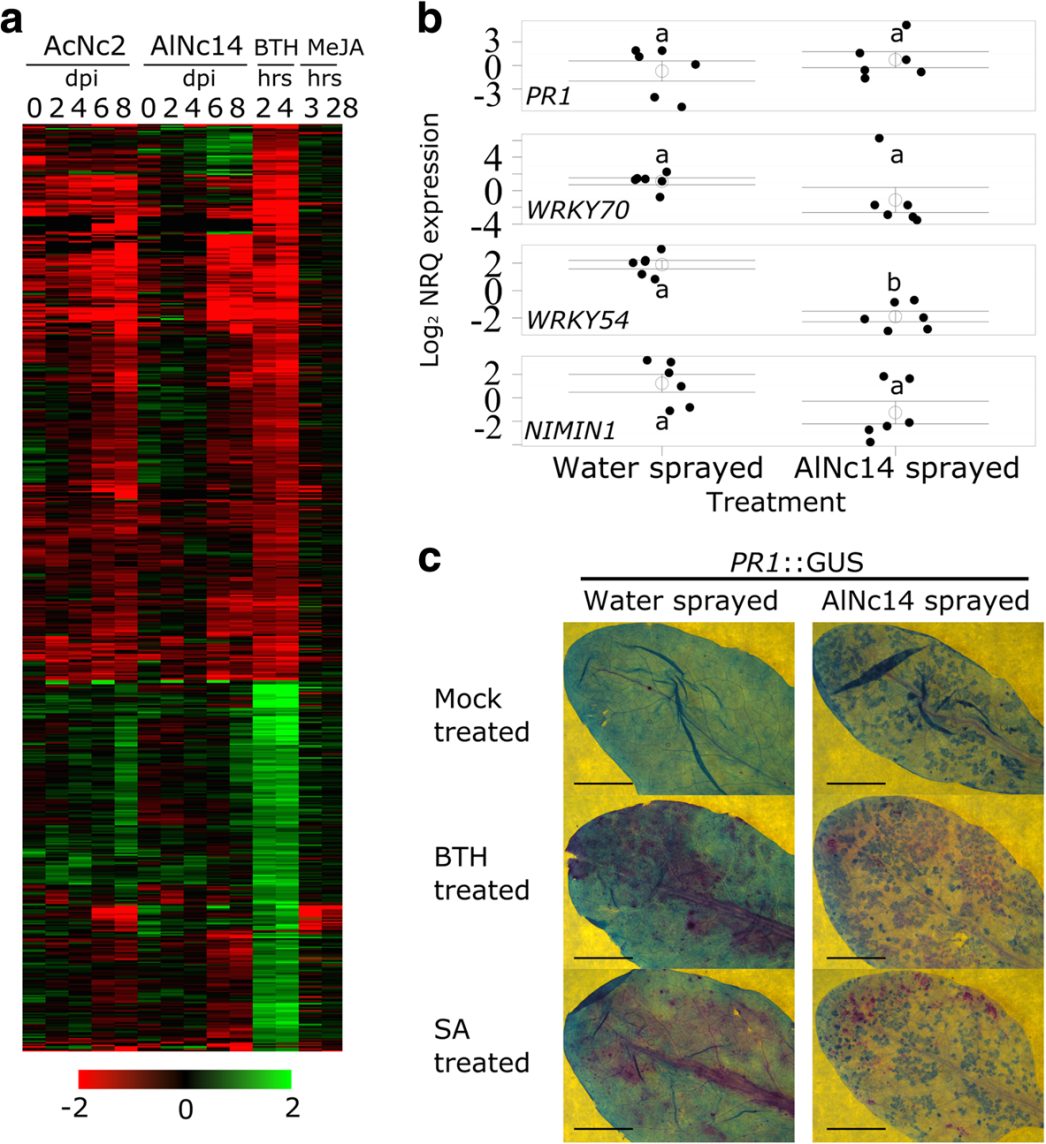
*Albugo*-infected leaves reveal reduced expression of salicylic acid (SA)-regulated genes. **a** Expression pattern of 671 benzo-(1,2,3)-thiadiazole-7-carbothioic acid (BTH)-inducible genes reported by [66] after inoculation with AcNc2 and AlNc14 over an 8-day time course in MAGIC 107. The data are the average of four experiments. The expression of the same genes during methyl jasmonate treatment [67, 68] are shown for comparison. The relative expression (in log_2_ ratios) is colored red for induction and green for repression as illustrated in the color bar. **b** Altered SA-regulated gene expression in AlNc14 infected Arabidopsis Col-0. Open circles and bars denote the mean ± SE of target gene expression (log_2_ transformed normalized relative quantities) in AlNc14 infected tissue from three independent biological replicates with two technical replicates per biological replicate. Closed, black circles denote the individual data points. Different letters indicate significant differences (*P* < 0.05) in gene expression (Welch Two Sample t-test (*PR1*, *P* = 0.395, *WRKY54*, *P* < 0.001, *NIMIN1*, *P* = 0.072), Wilcoxon rank sum test (*WRKY70*, *P* = 0.065) followed by Bonferroni correction). **c** AlNc14 suppresses BTH and SA induction of *PR1*. To visualize reporter gene induction and pathogen growth in the same leaf, leaves were collected and stained with magenta-GUS to reveal GUS activity, followed by trypan blue to reveal pathogen growth. Leaves of Col-0 pro(*PR1*)*::*GUS were previously inoculated with water or AlNc14 (13 dpi) and infiltrated with DMSO (mock), BTH (200 μM) or SA (200 μM) for 8 hours, then stained. Scale: 5 mm. Leaf images are from the same biological replicate and are representative of the average percentage of staining for each treatment across three independent biological replicates.

As depletion of tryptophan-derived secondary metabolites did not fully mimic the susceptibility of *Albugo*-infected plants to *P. infestans* we looked for additional candidate pathways in the GO enrichment analysis of the expression profiling. As previously noted, genes upregulated by both pathogens were enriched for GO terms associated with SA signaling (Table 1). To investigate this further, we visualized Arabidopsis genes differentially regulated by the SA mimic BTH [66] in our expression data (Fig. 8a, Additional file 22). The results showed a mixture of responses by BTH-regulated genes to *Albugo* infection, suggesting a subset of SA responsive genes may be targeted by the pathogens. In particular, a set of genes were less expressed during infection with either pathogen compared to BTH treatment (top of the figure). GO enrichment analysis of Arabidopsis genes differentially expressed specifically by AlNc14 also revealed SA biosynthesis and signaling to be down-regulated (Additional file 23).

### SA-regulated gene verification

To confirm the gene expression changes in *Albugo*-MAGIC 107 interactions mirrored those in *Albugo-*Col-0 interactions we conducted qRT-PCR on AlNc14-infected Col-0 Arabidopsis using a set of genes often used as SA markers (*PR1*, *non-inducible immunity1-interacting 1* (*NIMIN1*), *WRKY54* and *WRKY70* [36, 66, 94, 95]). These genes had different expression profiles over the time course of our data, with *PR1* being significantly up-regulated at 4 dpi and not differentially expressed at other time points, *WRKY54* being significantly down-regulated at 4, 6, and 8 dpi, *NIMIN1* being significantly down-regulated at 6 and 8 dpi, and *WRKY70* being significantly down-regulated at 8 dpi (Additional file 22). Using qRT-PCR we found that, at 10 dpi AlNc14, *WRKY54* was significantly down-regulated (*P* < 0.001), while *PR1* expression did not significantly change (*P* = 0.395), and *WRKY70* and *NIMIN1* showed non-significant trends of being down-regulated (*P =* 0.065 and *P* = 0.072, respectively) (Fig. 8b, Additional file 24). These data show similarities to the expression profile data, and therefore suggest that interactions between *Albugo* and MAGIC 107/Col-0 are likely to be similar.

Recent studies with *Hpa* have shown that the pathogen triggers *PR1* expression in the surrounding plant tissue while locally suppressing it in haustoriated cells [49, 50]. This cell-specific response is not captured in qRT-PCR assays of whole leaves. We used *PR1*::GUS promoter Arabidopsis line to explore whether AlNc14 suppresses *PR1* expression. We combined magenta-GUS staining with trypan blue staining to reveal both the reporter gene induction (purple) and the pathogen (dark blue). In striking contrast to *Hpa*, AlNc14 does not trigger high levels of *PR1* expression in surrounding tissue (Fig. 8c), suggesting suppression of immunity can be imposed systemically in non-haustoriated cells. We tested whether AlNc14 infection could suppress *PR1* induction in response to BTH and SA. Significantly more GUS expression was seen in water-pre-treated plants after BTH and SA treatment compared to AlNc14 pre-treated plants. The treatments that we compared were inoculation (water or AlNc14: F_(1, 74)_ = 21.65, *P* < 0.001), treatment (mock, BTH or SA: F_(1, 74)_ = 84.23, *P* < 0.001), and interaction between inoculation and treatment (F_(1, 74)_ = 45.72, *P* < 0.01; two-way ANOVA with Tukey’s HSD) (Fig. 8c, Additional files 5 and 13). Thus, these data show that AlNc14 can suppress the expression of some of the Arabidopsis genes induced by SA.

### SA signaling suppression is not sufficient for susceptibility of Arabidopsis to *P. infestans*

**Fig. 9:**
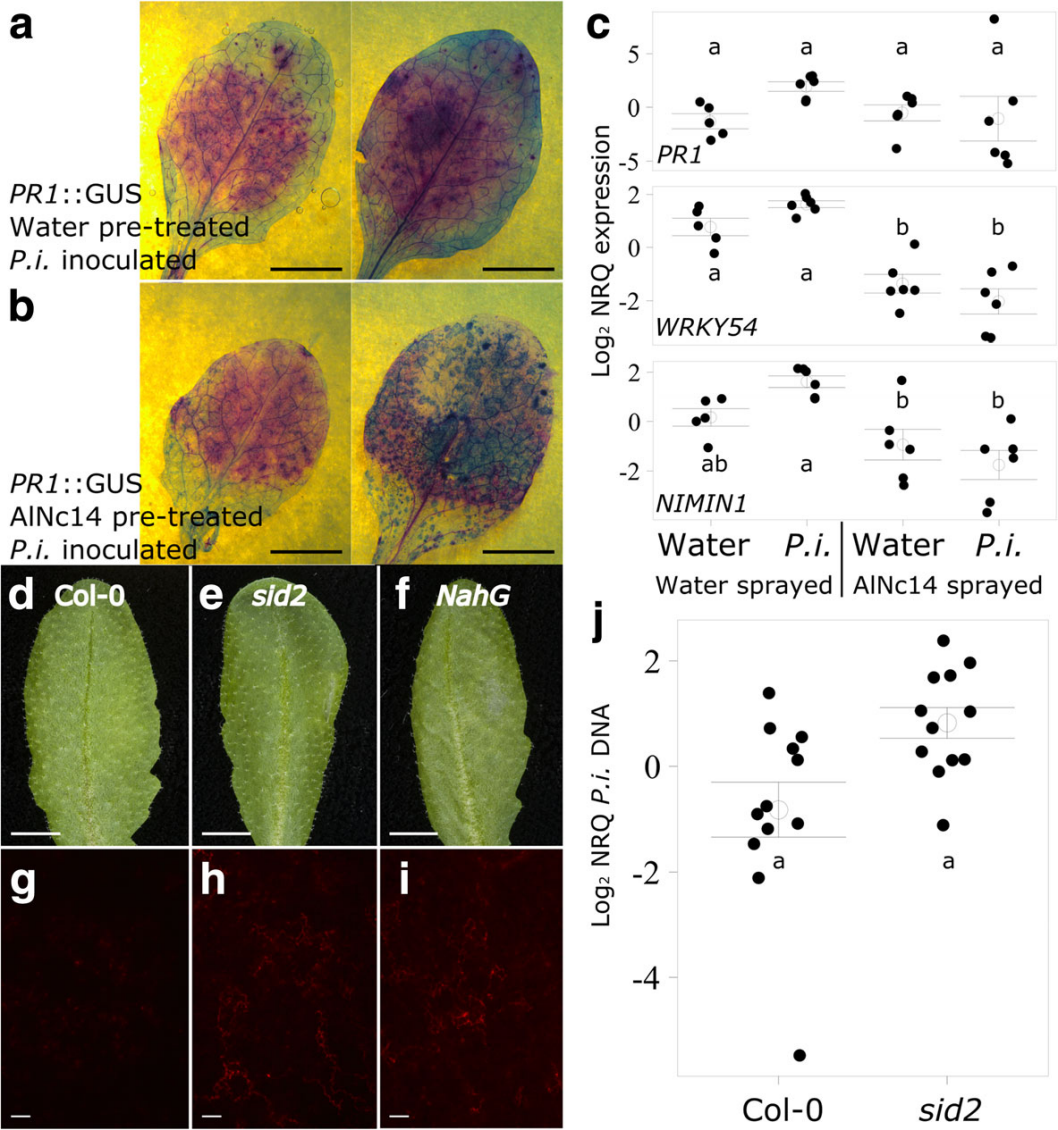
*Albugo* suppression of Arabidopsis salicylic acid (SA) responses is not sufficient for full susceptibility to *P. infestans*. **a** and **b** *PR1*::GUS staining upon *P. infestans* infection. Leaves were collected and stained with magenta-GUS to reveal GUS activity, followed by trypan blue to reveal pathogen growth. *PR1*::GUS plants were pre-treated with water or AlNc14 and subsequently inoculated with 100 μL of 1.25 × 10^5^ spores per mL *P. infestans* 88069td, collected at 2 dpi and stained. Scale: 5 mm. Representative leaves shown are from each of two independent experiments. **c** AlNc14 infection prevents *P. infestans*-induced upregulation of SA marker genes in Col-0. Open circles and bars denote the mean ± SE of target gene expression (log_2_ normalized relative quantities (NRQs)) at 48 hours post treatment (100 μL water or *P. infestans* (1.25 × 10^5^ spores per mL)) of three independent biological replicates with two technical replicates each. Closed, black circles denote the individual data points. Different letters indicate significant differences (*P* < 0.05; two-way ANOVA with Tukey’s HSD test). **d–i** *P. infestans* partially colonizes *sid2* and *NahG* Arabidopsis. Leaves were inoculated with 100 μL of 1 × 10^5^ spores per mL *P. infestans* 88069td, photographed (**d–f**) and the adaxial surface examined using fluorescence microscopy (**g–i**) at 3 dpi. Red fluorescence denotes *P. infestans* growth, Scale bars: 5 mm for photographs, 1 mm for microscopy. Results shown are representative of three independent experiments. **j** *P. infestans* growth on *sid2* is not significantly larger than Col-0 Arabidopsis. Leaves were inoculated as in **d**, **e**, **g**, **h**. DNA was extracted at 3 dpi and the proportion of *P. infestans* DNA to plant DNA determined using qRT-PCR. Open circles and bars denote the mean ± SE of *P. infestans* DNA (log_2_ transformed NRQs) in Arabidopsis tissue from four independent biological replicates with three technical replicates each. Closed, black circles denote the individual data points. The two genotypes were not significantly different (*P* = 0.012) (Wilcoxon rank sum test followed by Bonferroni correction)

We next explored whether the suppression of plant SA responses by AlNc14 occurred during the interaction with *P. infestans*, which has been shown to induce *PR1* expression at 2–3 dpi in Arabidopsis [16]. To see if AlNc14 suppresses *P. infestans-*induced *PR1* expression, we infected AlNc14 and water-sprayed *PR1*::GUS leaves with *P. infestans.* We did not observe the same decrease in magenta GUS staining in the *Albugo*-inoculated leaves compared to the water-sprayed leaves with *P. infestans* infection (Fig. 9a and b) that was seen for SA and BTH treatments. To further investigate potential suppression of SA responses to *P. infestans* in AlNc14-infected plants, we conducted qRT-PCR on SA marker genes *PR1*, *WRKY54*, and *NIMIN1* in leaves of AlNc14-infected or water-sprayed control plants that were subsequently drop inoculated with water or *P. infestans* (Fig. 9c, Additional file 25). *PR1* expression did not vary across the treatments (pre-treatment: F_(1, 19)_ = 1.066, *P* = 0.315; inoculation: F_(1, 19)_ = 1.075, *P* = 0.313; interaction: F_(1, 19)_ = 2.428, *P* = 0.136; two-way ANOVA). *WRKY54* expression was significantly decreased in AlNc14-infected leaves compared to water-sprayed control leaves (pre-treatment: F_(1, 19)_ = 71.520, *P* < 0.001; inoculation: F_(1, 19)_ = 0.026, *P* = 0.8738; interaction: F_(1, 19)_ = 4.796, *P* = 0.041; two-way ANOVA with Tukey’s HSD). *NIMIN1* expression was significantly decreased in AlNc14-infected leaves compared to *P. infestans* inoculated water-sprayed control leaves (pre-treatment: F_(1, 19)_ = 22.096, *P* < 0.001; inoculation: F_(1, 19)_ = 0.274, *P* = 0.607; interaction: F_(1, 19)_ = 5.327, *P* = 0.032; two-way ANOVA with Tukey’s HSD). In summary, we demonstrated that AlNc14 suppresses *P. infestans*-triggered *NIMIN1* expression and confirmed our previous finding that AlNc14 suppresses *WRKY54* expression.

*Isochorismate synthase 1* (*ics1*) (a.k.a. *SA-induction deficient 2* (*sid2*)) is required for SA biosynthesis, and *ics1* mutants accumulate very low levels of SA upon pathogen challenge [96]. Since *Albugo* infection suppresses some of the plant SA responses, we tested whether *sid2* was susceptible to *P. infestans*. Observations of infected *sid2* leaves showed small amounts of cell death in response to *P. infestans* infection (Fig. 9e). Microscopy revealed a greater degree of tissue colonization in *sid2* than Col-0 (Fig. 9g and h), although no *P. infestans* spore formation was observed. A similar phenotype of cell death and increased *P. infestans* colonization without spore formation was seen in the *NahG* Arabidopsis line (Fig. 9f and i) which expresses salicylate hydroxylase and degrades SA into catechol [97]. To quantify the amount of *P. infestans* biomass on *sid2* compared to Col-0 we estimated relative levels of *P. infestans* DNA using qRT-PCR (Fig. 9j, Additional file 25). Although a trend of increased *P. infestans* colonization of *sid2* was seen (*P* = 0.012), this was not statistically significant after Bonferroni correction. Taken together, these data suggest that *Albugo* can suppress a subset of SA responses in Arabidopsis, but the lack of SA responsiveness is unlikely to significantly contribute to the susceptibility of *Albugo*-infected Arabidopsis to *P. infestans*.

## Discussion

We investigated mechanisms of immuno-suppression by *Albugo* spp., in particular its remarkable capacity to render Arabidopsis susceptible to the potato late blight pathogen *P. infestans* [12]. Our data reveal alterations in tryptophan-derived secondary metabolite biosynthesis and availability, a role for tryptophan-derived secondary metabolites in Arabidopsis NHR to *P. infestans*, and suppression of host defense triggered by SA in *Albugo*-infected tissue.

Confirming that *A. candida* suppresses Arabidopsis NHR to *P. infestans* allowed us to use two *Albugo* species to investigate shared plant genes altered by *Albugo* infection through expression profiling. We saw a large number of differentially expressed plant genes between uninfected and infected tissue, which is in contrast to a recent study of the apoplastic proteome of uninfected and *A. laibachii*-infected tissue that found no significant differences [98]. Surprisingly, the only enriched GO terms in genes downregulated by both pathogens were photosynthesis, commonly downregulated in plants under biotic stress [99], and RNA elongation. The enriched GO terms in genes upregulated by both pathogens were generally related to plant defense responses (SA and JA), again surprising given the immuno-compromised nature of the host. Although cells colonized by haustoria may be completely immunosuppressed, adjacent cells may be the source of defense activation revealed in expression profiling, as seen with *Hpa* infection [49]. However, we cannot rule out the possibility that *Albugo* may cause changes in immunity at the protein level in addition to the level of the transcriptome. Changes in secondary metabolites common among *Albugo* hosts but absent from *P. infestans* hosts can be regarded as plausible candidates for a role in *P. infestans* NHR.

To investigate how *Albugo* might alter tryptophan-derived secondary metabolites, we measured gene expression and metabolite accumulation in response to *P. infestans* in the presence and absence of *Albugo*. Arabidopsis responds to *P. infestans* inoculation by upregulating the genes involved in camalexin biosynthesis, leading to camalexin accumulation. The main changes in the indolic glucosinolate pathway were an upregulation of *SOT16* at early time points and upregulation of *CYP81F2* at early and late time points, with no change in the accumulation of I3M and 4MO-I3M. Accumulation of camalexin and indolic glucosinolates in Arabidopsis in response to non-host pathogens is not uniform. Challenge with biotrophic *Bgh* leads to no change in camalexin, a decrease in I3M and no change in 4MO-I3M [18], whereas challenge with the necrotrophic fungus *Plectosphaerella cucumerina* and an incompatible strain of *P. brassicae* leads to an increase in camalexin, a decrease in I3M, and an increase in 4MO-I3M [32, 100]. Responses to *P. infestans* in *Albugo-*infected Arabidopsis were similar to those in plants without *Albugo*, with the main difference being no significant *SOT16* expression and a significant reduction in I3M. The inability to separate I3M from other indole-3-acetaldoxime-derived indolic compounds makes it difficult to test with Arabidopsis mutants whether a reduction in I3M but not camalexin contributes to *P. infestans* NHR. *CYP83B1* mutants accumulate increased indole-3-acetic acid, resulting in pleiotropic effects (e.g., [101, 102]), whereas *SOT16* mutants are yet to be characterized but may also have a similar phenotype. *35S:DWF4* has reduced I3M compared to Col-0 and similar amounts of 4MO-I3M [89], but we found that this plant line was not susceptible to *P. infestans* in the absence of *Albugo* and was less susceptible than Col-0 in the presence of *Albugo*. While the transcriptional responses to *P. infestans* were similar in uninfected and *Albugo*-infected tissue, the response per amount of *P. infestans* was much lower in the *Albugo*-infected tissue due to increased *P. infestans* colonization in this tissue.

*cyp79b2/b3* is deficient in tryptophan-derived secondary metabolites including indolic glucosinolates and camalexin [103, 104] and is the first Arabidopsis mutant, to our knowledge, on which *P. infestans* can sporulate, if only occasionally. As the *pen2-1 pad3* mutant, deficient in camalexin and hydrolysis of 4MO-I3M, did not show the same level of *P. infestans* colonization as *cyp79b2/b3*, we conclude that tryptophan-derived antimicrobial metabolites, in addition to camalexin and indolic glucosinolates, play a role in *P. infestans* NHR in Arabidopsis. Our data agree with recent reports [32, 100, 105] of uncharacterized tryptophan-derived secondary metabolites that play an important role in immunity to non-adapted filamentous pathogens. The recent discovery that Arabidopsis synthesizes 4-hydroxyindole-3-carbonyl nitrile from tryptophan, and that mutants in its biosynthesis are more susceptible to the hemibiotroph bacterial pathogen *Pseudomonas syringae* [106], emphasizes that other molecules contributing to plant defense may remain to be discovered.

*Albugo*-infected *cyp79b2/b3* mutants support more *P. infestans* growth than uninfected *cyp79b2/b3*, suggesting that either *Albugo*-infection has a stronger phenotype than the *cyp79b2/b3* mutant, or mechanisms in addition to indole glucosinolates, camalexin, and tryptophan-derived metabolites contribute to *P. infestans* resistance, and that these mechanisms are also suppressed by *Albugo* infection. The *Albugo-*infected mutant was not more susceptible than infected Col-0, suggesting that indole-derived metabolites are less effective at suppressing microbial growth in *Albugo*-infected plant tissue. If *Albugo* suppression of NHR was working separately to tryptophan-derived secondary metabolites, then we would expect that *Albugo-*infected plants of *cyp79b2/b3* would show additional enhanced susceptibility compared to *Albugo-*infected Col-0. This suggests that there is interplay between NHR and tryptophan-derived secondary metabolites, although conceivably the additive phenotype was overlooked due to technical limitations. In addition to tryptophan-derived secondary metabolites, we also identified a very minor role for SATI in Arabidopsis NHR to *P. infestans*, but it is possible that other aspects of plant immunity contribute too.

*Albugo-*infected plants accumulate camalexin in the absence and presence of *B. cinerea.* However, both wild type *B. cinerea* and the camalexin-sensitive mutant ΔBcatrB4 produce bigger lesions on *Albugo*-infected plants, while the BcatrBp803GUS-7 *B. cinerea* strain responds as if the amount of camalexin in *Albugo*-infected plants is the same as in a camalexin-deficient *pad3* mutant. We therefore conclude that the camalexin must be biologically unavailable to *B. cinerea*, and also possibly to *P. infestans*. How camalexin is made biologically unavailable remains to be determined. Conceivably, *Albugo* infection leads to the compartmentalization of camalexin away from *B. cinerea* and other pathogens potentially accumulated within the *Albugo* cells. Alternatively, camalexin may be modified by *Albugo* in some way to make it biologically inert, though no such modification is visible in our metabolomics analysis. A recent study demonstrated that metabolites inhibiting the germination of *P. infestans* spores required secreting to the leaf surface to be effective [107]; therefore, it is also possible that *Albugo* alters metabolite transport, and hence spatial distribution. Whether altering tryptophan-derived metabolite biosynthesis and availability provides an advantage to *Albugo*, and is a direct result of *Albugo* effectors, remains unresolved. Some pathogens, such as the maize smut fungus *Ustilago maydis*, use effectors to manipulate plant metabolism to their advantage [108, 109]. Other pathogens have been shown to detoxify plant phytoalexins by active transport [51] or enzymatic modification [33–35]. Tryptophan-derived secondary metabolites are unlikely to be essential for *Albugo* infection of Arabidopsis, as *Albugo* can infect *cyp79b2/b3* and reduce NHR to *P. infestans* to the same extent as Col-0.

We also investigated SA-responsive gene expression in *Albugo*-infected tissue. We conducted qRT-PCR to investigate the expression of four SA marker genes identified in the expression profiling. The qRT-PCR largely matched the expression profiling, with *WRKY54* being significantly down-regulated, *WRKY70* and *NIMIN1* showing less expression, and *PR1* showing no change. We also used *PR1*::GUS reporter lines and SA/BTH to show that *Albugo* suppresses *PR1*::GUS transcription in the presence of SA/BTH. The suppression of SATI by *Albugo* provides a potential explanation for the observation that *A. laibachii* colonization is not significantly increased on *sid2* compared with Col-0 [98], and may also partly explain the impairment of host resistance against other pathogens [10, 11]. We have proposed that defense suppression is not only necessary for the pathogen’s own colonization, but also may allow different isolates to co-exist on a common host in order to facilitate hybridization between races that would not otherwise colonize the same host [10].

*P. infestans* induces expression of *PR1*::GUS in Arabidopsis [16]. *Albugo*-infected Arabidopsis does not show the clear suppression of *PR1*::GUS expression upon *P. infestans* challenge that was seen with BTH and SA. SA marker gene expression was not significantly induced in our qRT-PCR experiments with *P. infestans*. This may be because expression is localized to the site of inoculation, therefore being diluted at the whole leaf level, or the level of expression induced by *P. infestans* is relatively small. Alternatively, a more frequent time course experiment could be conducted to identify whether these genes peak in expression. *NIMIN1* was significantly down-regulated upon *P. infestans* challenge in *Albugo*-infected tissue compared to uninfected tissue, thus providing evidence that SATI to *P. infestans* is compromised in the presence of *Albugo*. Arabidopsis mutants in SATI are more susceptible to *P. capsici* [31]. A slight decrease in resistance, e.g., trailing necrosis, was also observed upon infection of *NahG* and *nonexpresser of pr genes 1* (*npr1*) plants after inoculation with an incompatible strain of *P. brassicae* [110]. The SA biosynthesis mutant *sid2* supported more *P. infestans* colonization compared to Col-0. Our results differ from a recent report of *P. infestans* infection of *sid2*, which did not identify any increase in *P. infestans* colonization or any increased cell death compared to Col-0 [25]. This may be due to a difference in the *P. infestans* strains used or the conditions for the experiments. We did not observe *P. infestans* spore formation on *sid2* Arabidopsis, unlike *Albugo*-infected tissue and *cyp79b2/b3*. This suggests that the contribution of SATI to *P. infestans* NHR is likely to be minor.

## Conclusions

Previously, *Albugo* suppression of plant immunity had been described but the mechanisms involved had not been investigated. Now, the identification of *Albugo*-induced alterations in tryptophan-derived secondary metabolite biosynthesis and availability and suppression of SATI will inform more focused studies on potential *Albugo* effectors, as for other pathogens and pests [111, 112], by providing phenotypes to screen for. Identification of proteins that are recognized by plants, leading to resistance against *Albugo* will also help identify likely effectors. In the future, it may be possible to take advantage of the apparent conservation of function of secondary metabolites in plant immunity [27] by using tryptophan-derived secondary metabolites and other phylogenetically limited metabolites in crop protection strategies against *P. infestans* and other pathogens or pests, either through direct application of the metabolites or by transgenically transferring the metabolic pathways into new species.

## Additional files

Additional file 1: Plant lines used in the study. A list of the Arabidopsis ecotypes, crossed lines, mutants and transgenic lines used in the study. All mutants and transgenic lines are in the Col-0 background except *pen2-1* and Col-gl *RPW8.1 RPW8.2*, which are in the *glabrous1* background. (DOCX 18 kb)
Additional file 2: Arabidopsis genes differently regulated in expression profiling through randomly sheared cDNA tag sequencing (EXPRSS) data. Lists of the Arabidopsis genes that were differentially expressed in AlNc2 and AlNc14 infected tissue over a time course. (XLSX 1019 kb)
Additional file 3: Differentially regulated genes at each time point and overlap between genes regulated by each pathogen. Comparisons of the Arabidopsis genes differentially expressed during infection with AlNc2 and/or AlNc14 over a time course. (XLSX 104 kb)
Additional file 4: Primers used in the study. Details of the primers used to conduct qRT-PCR in the study. (DOCX 25 kb)
Additional file 5: AlNc14 suppresses benzo-(1,2,3)-thiadiazole-7-carbothioic acid (BTH) and salicylic acid (SA) induction of *PR1.* To visualize reporter gene induction and pathogen growth in the same leaf, leaves were collected and stained with magenta-GUS to reveal GUS activity, followed by trypan blue to reveal pathogen growth. Leaves of Col-0 pro(*PR1*)*::*GUS were previously inoculated with water or AlNc14 (13 dpi) and infiltrated with DMSO (mock), BTH (200 μM) or SA (200 μM) for 8 hours, then stained and examined with a microscope. The percentage of each leaf stained with GUS was determined using ImageJ. (A) Open circles represent mean ± SE of the raw data (percentage of leaf stained) of three independent biological replicates with between two and seven technical replicates per biological replicate (bars left to right n = 10, 12, 13, 15, 14 and 16). (B) Open circles represent mean ± SE of the transformed data (arcsine square root transformation followed by log_10_ transformation) of three independent biological replicates with between two and seven technical replicates per biological replicate (bars left to right n = 10, 12, 13, 15, 14 and 16). Different letters indicate significant differences *P* < 0.001 (Two-way ANOVA, Tukey’s HSD test). (PDF 33 kb)
Additional file 6:
*Hyaloperonospora arabidopsidis* (*Hpa*) Waco9 infection does not allow *P. infestans* colonization of Arabidopsis. (A) Water sprayed, (B) AlNc14 sprayed (12 dpi) and (C) *Hpa* sprayed leaves (6 dpi) were drop inoculated with 100 μL of 3.25 to 5 × 10^4^ spores per mL *P. infestans* 88069td. Fluorescence microscopy of the adaxial surface of the leaf taken at 3 dpi *P. infestans*. Scale bar: 100 μm. Results shown are representative of two independent experiments. (TIF 3605 kb)
Additional file 7: Gene ontology (GO) terms within biological processes that are significantly enriched amongst genes up-regulated in the expression profiling. Results of the GO enrichment analysis for up-regulated genes. (XLSX 328 kb)
Additional file 8: Gene ontology (GO) terms within biological processes that are significantly enriched amongst genes down-regulated in the expression profiling. Results of the GO enrichment analysis for down-regulated genes. (XLSX 157 kb)
Additional file 9: Two-way ANOVA results from qRT-PCR of tryptophan-derived secondary metabolite genes at 6 hours post *P. infestans* inoculation. ANOVA table. (DOCX 13 kb)
Additional file 10: Data analyzed in Fig. 4. Spreadsheets showing the data analyzed in Fig. 4a–d. (XLSX 30 kb)
Additional file 11: Two-way ANOVA results from qRT-PCR of tryptophan-derived secondary metabolite genes at 48 hours post *P. infestans* inoculation. ANOVA table. (DOCX 13 kb)
Additional file 12: Indolic glucosinolate measurements in *pen2-1* plants in response to pre-treatment with water or *Albugo* and subsequent inoculation with water or *P. infestans*. HPLC analysis of mock or *Albugo* infected *pen2-1* tissue (12 dpi), 20 hours post mock or *P. infestans* treatment (100 μL of 3 × 10^5^ spores per mL). Open circles and bars denote mean indolic glucosinolate content ± SE of three independent biological replicates with six technical replicate per biological replicate. Closed, black circles denote the individual data points. Different letters indicate significant different values within each glucosinolate measured (*P* < 0.05) (Two-way ANOVA, Tukey’s HSD test). (PDF 175 kb)
Additional file 13: Data analyzed in Additional files. Spreadsheets showing the data analyzed in Additional files 11, 16 and 20. (XLSX 27 kb)
Additional file 14:
*P. infestans* forms haustoria in *cyp79b2/b3* plants. Leaves of *Nicotiana benthamiana* (A and B) and Arabidopsis *cyp79b2/b3* (C and D) were drop inoculated with 50 μL of 1 × 10^5^ spores per mL *P. infestans* 88069td and examined using confocal microscopy at 2 dpi (A and B) and 3 dpi (C and D). A and C show colonization of the leaf by *P. infestans*. Scale bar = 100 μM. B and D show formation of infection structures by *P. infestans*, with haustoria denoted by asterisks. Scale bar = 10 μM. (PDF 485 kb)
Additional file 15:
*P. infestans* sporulation on *cyp79b2/b3* plants. Leaves of Col-0 and *cyp79b2/b3* were inoculated with 100 μL of 2.5 × 10^5^ spores per mL *P. infestans* NL12226. Photographs were taken of the abaxial surface of Col-0 (A) and *cyp79b2/b3* (B) leaves at 3 dpi. Scale bars: 5 mm. (C and D) Leaves were examined for sporulation between 3 and 5 dpi by placing water droplets on the leaves and examining them for the presence of spores using a light microscope (C). Frequency of sporulating leaves in three independent experiments were recorded (D) (Replicate 1: Col-0, n = 44, sporulating = 0; *cyp79b2/b3*, n = 56, sporulating = 5. Replicate 2: Col-0, n = 42, sporulating = 0; *cyp79b2/b3*, n = 62, sporulating = 4. Replicate 3: Col-0, n = 22, sporulating = 0; *cyp79b2/b3*, n = 71, sporulating = 0). (TIF 9710 kb)
Additional file 16:
*Albugo-*infected *35S:DWF4* is less susceptible to *P. infestans* than *Albugo-*infected Col-0*.* (A–D) Fluorescence microscopy of the adaxial surface of water-sprayed Col-0 (A), water-sprayed *35S:DWF4* (B), *Albugo*-infected Col-0 (C) and *Albugo*-infected *35S:DWF4* (D) leaves. Leaves were sprayed with water or *Albugo* and subsequently inoculated (12 days post spraying) with 100 μL of 1 × 10^5^ spores per mL *P. infestans* 88069td. Leaves were examined using fluorescence microscopy at 3 dpi. Red fluorescence denotes *P.i.* growth. Scale bars: 200 μm. Results shown are representative of three independent experiments. (E and F) Photographs of *Albugo*-infected Col-0 (E) and *Albugo*-infected *35S:DWF4* (F) leaves, infected as described above, were taken at 3 dpi. Scale bars: 5 mm. (G) Quantification of *P. infestans* biomass on *Albugo* infected Col-0 and *35S:DWF4* by qRT-PCR. Leaves were inoculated with 100 μL of 1 × 10^5^ spores per mL *P. infestans* 88069td. DNA was extracted at 3 dpi and the proportion of *P. infestans* DNA to plant DNA determined using qRT-PCR. Open circles and bars denote means ± SE of three independent biological replicates with three technical replicates per biological replicate. Closed, black circles denote the individual data points. Different letters indicate significant differences (Welch two sample t-test) (*P* < 0.001). (TIF 3282 kb)
Additional file 17: Data analyzed in Fig. 6. Spreadsheets showing the data analyzed in Fig. 6a and b. (XLSX 17 kb)
Additional file 18:
*myb28/29* and *tgg1/tgg2* are not susceptible to *P. infestans*. (A–C) Leaves of Col-0, *myb28/29* and *cyp79b2/b3* (positive control) were inoculated with 100 μL of at least 1 × 10^5^ spores per mL *P. infestans* 88069td. (D–F) Leaves of Col-0, *tgg1/2* and AlNc14 sprayed Col-0 (positive control) were inoculated with 100 μL of at least 1 × 10^5^ spores per mL *P. infestans* 88069td. The adaxial surface of the leaves was examined using fluorescence microscopy at 3 dpi. Scale bars: 100 μm. Red fluorescence denotes *P.i.* growth. Results shown are representative of two (A–C) and three (D–F) independent experiments. (TIF 5249 kb)
Additional file 19: Data analyzed in Fig. 7. Spreadsheets showing the data analyzed in Fig. 7a–c. (XLSX 17 kb)
Additional file 20: Example of staining of GUS-expressing *B. cinerea* strains on water and *Albugo-*sprayed Col-0 leaves. Photograph of three representative leaves. Top row are AlNc14-infected leaves and the bottom row are water-sprayed leaves. The left hand side of each leaf received three droplets of OliCGUS *B. cinerea* and the right hand side received three drops of BcatrBp803GUS-7 *B. cinerea*. Leaves were removed from the plant and stained at 2 dpi *B. cinerea. (TIF 6351 kb)*

Additional file 21:
*B. cinerea* detects less available camalexin in *Albugo*-infected tissue. Leaves underwent protein extraction and GUS enzyme activity was determined using a fluorescence-based assay. Results were normalized to *B. cinerea* weight proportion of each sample using qRT-PCR on Botrytis and Arabidopsis genomic DNA. Open circles and bar dots represent the mean ± SE of three independent biological replicates with three or four technical replicates per biological replicate. Closed, black circles denote the individual data points. Asterisk indicates significant differences measured at *P* < 0.05 (Wilcoxon rank sum test within *B. cinerea* strain followed by Bonferroni correction), n.s. = not significant. (PDF 176 kb)
Additional file 22: Benzo-(1,2,3)-thiadiazole-7-carbothioic acid (BTH) regulated genes during *Albugo* infection time course. Spreadsheet showing the expression of Arabidopsis BTH regulated genes during infection with *Albugo*. (XLSX 346 kb)
Additional file 23: List of selected, lower level gene ontology (GO) terms enriched in genes differentially expressed during AlNc14 infection but not AcNc2 infection. Table showing the GO terms enriched in Arabidopsis genes differentially expressed during AlNc14 infection only. (DOCX 14 kb)
Additional file 24: Data analyzed in Fig. 8. Spreadsheets showing the data analyzed in Fig. 8b. (XLSX 14 kb)
Additional file 25: Data analyzed in Fig. 9. Spreadsheets showing the data analyzed in Fig. 9c and j. (XLSX 16 kb)

## Abbreviations

4MO-I3M: 4-methoxyindol-3-ylmethylglucosinolate
ABC: ATP-binding cassette
Ac: *Albugo candida*
ANOVA: analysis of variance
BTH: benzo-(1,2,3)-thiadiazole-7-carbothioic acid
CER: controlled environment room
CYP: cytochrome P450
dpi: days post inoculation
DWF4: dwarf 4
EDTA: ethylenediaminetetraacetic acid
ETI: effector-triggered immunity
EXPRSS: expression profiling through randomly sheared cDNA tag sequencing
FDR: false discovery rate
GLM: generalized linear model
GO: gene ontology
GUS: β-glucuronidase
Hpa: hyaloperonospora arabidopsidis
hpi: hours post inoculation
HPLC: high performance liquid chromatography
HSD: honest significant difference
I3M: indol-3-ylmethylglucosinolate
JA: jasmonic acid
MAGIC: multiparent advanced generation inter-cross
NHR: non-host resistance
NIMIN1: non-inducible immunity1-interacting 1
NRQs: normalized relative quantities
PAD3: phytoalexin deficient 3
PEN: penetration
PR1: pathogenesis-related 1
PTI: pattern-triggered immunity
SA: salicylic acid
SATI: SA-triggered immunity
SOT16: sulfotransferase 16
TAIR10: The Arabidopsis Information Resource version 10.
