# Supplementary material for: Albugo-imposed changes to tryptophan-derived antimicrobial metabolite biosynthesis may contribute to suppression of non-host resistance to Phytophthora infestans in Arabidopsis thaliana
Source: BMC Biol. 2017 Mar 20;15:20. doi: 10.1186/s12915-017-0360-z (PMC5358052; doi:10.1186/s12915-017-0360-z)
Supplement: Additional file 1: — Plant lines used in the study. A list of the Arabidopsis ecotypes, crossed lines, mutants and transgenic lines used in the study. All mutants and transgenic lines are in the Col-0 background except pen2-1 and Col-gl RPW8.1 RPW8.2, which are in the glabrous1 background. (DOCX 18 kb) [file 12915_2017_360_MOESM1_ESM.docx]

**Additional file 1. Plant lines used in the study**

| **Ecotypes** | **Reference** |
| --- | --- |
| Col-0 |  |
| Ws-2 |  |
| **Crossed line** |  |
| MAGIC 107 | [1] |
| **Mutant plant lines** |  |
| *cyp79b2/b3* | [2] |
| *cyp81f2-1* | [3] |
| *myb28/29* | [4] |
| *pad3* | [5] |
| *pen2-1* | [6] |
| *pen2-1 pad3* | [3] |
| *sid2* | [7] |
| *tgg1 tgg2* | [8] |
| **Transgenic plant lines** |  |
| *35S:DWF4* | [9] |
| *NahG* | [10] |
| *PR1*::GUS | [11] |
| Col-gl *RPW8.1 RPW8.2* | [12] |

All mutants and transgenic lines are in the Col-0 background except *pen2-1* and Col-gl *RPW8.1 RPW8.2*, which are in the *glabrous1* background.

References

1. Kover PX, Valdar W, Trakalo J, Scarcelli N, Ehrenreich IM, Purugganan MD, et al. A multiparent advanced generation inter-cross to fine-map quantitative traits in *Arabidopsis thaliana*. PLoS Genet. 2009;5(7):e1000551.

2. Zhao Y, Hull AK, Gupta NR, Goss KA, Alonso J, Ecker JR, et al. Trp-dependent auxin biosynthesis in *Arabidopsis*: involvement of cytochrome P450s CYP79B2 and CYP79B3. Genes & Development. 2002;16(23):3100-12.

3. Bednarek P, Piślewska-Bednarek M, Svatoš A, Schneider B, Doubský J, Mansurova M, et al. A glucosinolate metabolism pathway in living plant cells mediates broad-spectrum antifungal defense. Science. 2009;323(5910):101-6.

4. Sønderby IE, Hansen BG, Bjarnholt N, Ticconi C, Halkier BA, Kliebenstein DJ. A systems biology approach identifies a R2R3 MYB gene subfamily with distinct and overlapping functions in regulation of aliphatic glucosinolates. PLoS ONE. 2007;2(12):e1322.

5. Glazebrook J, Ausubel FM. Isolation of phytoalexin-deficient mutants of *Arabidopsis thaliana* and characterization of their interactions with bacterial pathogens. Proceedings of the National Academy of Sciences. 1994;91(19):8955-9.

6. Lipka V, Dittgen J, Bednarek P, Bhat R, Wiermer M, Stein M, et al. Pre- and postinvasion defenses both contribute to nonhost resistance in *Arabidopsis*. Science. 2005;310(5751):1180-3.

7. Nawrath C, Métraux J-P. Salicylic acid induction–deficient mutants of Arabidopsis express *PR-2* and *PR-5* and accumulate high levels of camalexin after pathogen inoculation. The Plant Cell. 1999;11(8):1393-404.

8. Barth C, Jander G. Arabidopsis myrosinases TGG1 and TGG2 have redundant function in glucosinolate breakdown and insect defense. The Plant Journal. 2006;46(4):549-62.

9. Belkhadir Y, Jaillais Y, Epple P, Balsemão-Pires E, Dangl JL, Chory J. Brassinosteroids modulate the efficiency of plant immune responses to microbe-associated molecular patterns. Proceedings of the National Academy of Sciences. 2012;109(1):297-302.

10. Lawton K, Weymann K, Friedrich L, Vernooij B, Uknes S, Ryals J. Systemic acquired resistance in *Arabidopsis* requires salicylic acid but not ethylene. Molecular Plant-Microbe Interactions. 1995;8(6):863-70.

11. Caillaud M-C, Asai S, Rallapalli G, Piquerez S, Fabro G, Jones JDG. A downy mildew effector attenuates salicylic acid–triggered immunity in Arabidopsis by interacting with the host mediator complex. PLoS Biology. 2013;11(12):e1001732.

12. Xiao S, Ellwood S, Calis O, Patrick E, Li T, Coleman M, et al. Broad-spectrum mildew resistance in *Arabidopsis thaliana* mediated by *RPW8*. Science. 2001;291(5501):118-20.
