# Supplementary material for: Albugo-imposed changes to tryptophan-derived antimicrobial metabolite biosynthesis may contribute to suppression of non-host resistance to Phytophthora infestans in Arabidopsis thaliana
Source: BMC Biol. 2017 Mar 20;15:20. doi: 10.1186/s12915-017-0360-z (PMC5358052; doi:10.1186/s12915-017-0360-z)
Supplement: Additional file 4: — Primers used in the study. Details of the primers used to conduct qRT-PCR in the study. (DOCX 25 kb) [file 12915_2017_360_MOESM4_ESM.docx]

**Additional file 4. Primers used in the study**

| **Gene name** | **Identifier** | **Use in study** | **Sequence (5’>3’)** | **Reference** | **Annealing temperature used** |
| --- | --- | --- | --- | --- | --- |
| *RNA-BINDING (RRM/RBD/RNP MOTIFS) FAMILY PROTEIN* | At3g21215 | qRT-PCR reference gene | F GAATCCACCCATACCACCAG  R GAGGAGGAGGATGGTGATGA | [1] | 62 |
| O8 | PiO8-3-3 | qRT-PCR target gene | F CAATTCGCCACCTTCTTCGA  R GCCTTCCTGCCCTCAAGAAC | [2] | 62 |
| *SHAGGY-RELATED KINASE 11* | At5g26751 | qRT-PCR reference gene | F CTTATCGGATTTCTCTATGTTTGGC  R GAGCTCCTGTTTATTTAACTTGTACATACC | [3] | 60 |
| *CUTINASE A* | Z69264 | qRT-PCR target gene | F AGCCTTATGTCCCTTCCCTTG  R GAAGAGAAATGGAAAATGGTGAG | [3] | 60 |
|  |  |  |  |  |  |
| *EF1 ALPHA* | At5g60390 | qRT-PCR reference gene | F CAGGCTGATTGTGCTGTTCTTA  R GTTGTATCCGACCTTCTTCAGG | [4] | 62 |
| *TIP41* | At4g34270 | qRT-PCR reference gene | F TCCATCAGTCAGAGGCTTCC  R AAGAAAGCTCATCGGTACGC | [5] | 62 |
| *U-BOX* | At5g15400 | qRT-PCR reference gene | F TGCGCTGCCAGATAATACACTATT  R TGCTGCCCAACATCAGGTT | [6] | 62 |
| *GLYCERALDEHYDE-3-PHOSPHATE DEHYDROGENASE C2* | At1g13440 | qRT-PCR reference gene | F AGGTCAAGCATTTTCGATGC  R AACGATAAGGTCAACGACACG | [5] | 62 |
| ACTIN2 | At3g18780 | qRT-PCR reference gene | F GATGAGGCAGGTCCAGGAATC  R GTTTGTCACACACAAGTGCATC | [7] | 62 |
| *PEROXIN4* | At5g25760 | qRT-PCR reference gene | F TGCAACCTCCTCAAGTTCG  R CACAGACTGAAGCGTCCAAG | [7] | 62 |
| *MONENSIN SENSITIVITY1* | At2g28390 | qRT-PCR reference gene | F TGTGCCAAAGGGTAAAAGATG  R AGCACAATATAGGGGGTCAAAC | This study | 62 |
| *ADAPTOR PROTEIN-2 MU-ADAPTIN* | At5g46630 | qRT-PCR reference gene | F TGCGTTTTGGTTAATCTGTCTC  R CCGTGTTGTAACCGCTCTTC | [7] | 62 |
| *PR1* | At2g14610 | qRT-PCR target gene | F ATGAATTTTACTGGCTATTCTC  R AGGGAAGAACAAGAGCAACTA | [4] | 55.5 |
| *WRKY54* | At2g40750 | qRT-PCR target gene | F GCACTGCTCAGAACCATGTCAA  R CAAGTCCTCACCTGTCGAAGA | [8] | 62 |
| *WRKY70* | At3g56400 | qRT-PCR target gene | F CATGGATTCCGAAGATCACA  R CTGGCCACACCAATGACAA | [8] | 62 |
| *NIMIN1* | At1g02450 | qRT-PCR target gene | F CACGGAAACGTAGACGAGAA  R CCCGTACGACACTGAGAGAA | [9] | 62 |
| *CYP79B2* | At4g39950 | qRT-PCR target gene | F TCTCCGGTTTATCTCGTTCAGTA  R CGTGTCTCATTCTCAGGTAGCTT | [7] | 62 |
| *CYP71A13* | At2g30770 | qRT-PCR target gene | F TAAAGAGGTGCTTCGGTTGC  R TATCGCAGTGTCTCGTTGGA | [10] | 62 |
| *PAD3* | At3g26830 | qRT-PCR target gene | F TGCTCCCAAGACAGACAATG  R GTTTTGGATCACGACCCATC | [10] | 62 |
| *CYP83B1* | At4g31500 | qRT-PCR target gene | F TGCTGGTAGATATGGCGTGAC  R AAGGGACCCGAATATTAAACATC | [7] | 62 |
| *SOT16* | At1g74100 | qRT-PCR target gene | F CGAAGTCGTCGAACTCACAGAGTT  R AAAGACCTTCGAGGAGACATTCTTG | [11] | 62 |
| *CYP81F2* | At5g57220 | qRT-PCR target gene | F AATGGAGAGAGCAACACAATG  R ATACTGAGCATGAGCCCTTTG | [12] | 62 |

References

1. Weßling R, Panstruga R. Rapid quantification of plant-powdery mildew interactions by qPCR and conidiospore counts. Plant Methods. 2012;8(1):35.

2. Eschen-Lippold L, Rothe G, Stumpe M, Göbel C, Feussner I, Rosahl S. Reduction of divinyl ether-containing polyunsaturated fatty acids in transgenic potato plants. Phytochemistry. 2007;68(6):797-801.

3. Gachon C, Saindrenan P. Real-time PCR monitoring of fungal development in *Arabidopsis thaliana* infected by *Alternaria brassicicola* and *Botrytis cinerea*. Plant Physiology and Biochemistry. 2004;42(5):367-71.

4. Asai S, Rallapalli G, Piquerez SJM, Caillaud M-C, Furzer OJ, Ishaque N, et al. Expression profiling during Arabidopsis/downy mildew interaction reveals a highly-expressed effector that attenuates responses to salicylic acid. PLoS Pathogens. 2014;10(10):e1004443.

5. Czechowski T, Stitt M, Altmann T, Udvardi MK, Scheible W-R. Genome-wide identification and testing of superior reference genes for transcript normalization in Arabidopsis. Plant Physiology. 2005;139(1):5-17.

6. Wu X, Chory J, Weigel D. Combinations of *WOX* activities regulate tissue proliferation during *Arabidopsis* embryonic development. Developmental Biology. 2007;309(2):306-16.

7. Kettles GJ, Drurey C, Schoonbeek H-j, Maule AJ, Hogenhout SA. Resistance of *Arabidopsis thaliana* to the green peach aphid, *Myzus persicae*, involves camalexin and is regulated by microRNAs. New Phytologist. 2013;198(4):1178-90.

8. Besseau S, Li J, Palva ET. WRKY54 and WRKY70 co-operate as negative regulators of leaf senescence in *Arabidopsis thaliana*. Journal of Experimental Botany. 2012;63(7):2667-79.

9. Wang S, Durrant WE, Song J, Spivey NW, Dong X. *Arabidopsis* BRCA2 and RAD51 proteins are specifically involved in defense gene transcription during plant immune responses. Proceedings of the National Academy of Sciences. 2010;107(52):22716-21.

10. Nafisi M, Goregaoker S, Botanga CJ, Glawischnig E, Olsen CE, Halkier BA, et al. *Arabidopsis* cytochrome P450 monooxygenase 71A13 catalyzes the conversion of indole-3-acetaldoxime in camalexin synthesis. The Plant Cell. 2007;19(6):2039-52.

11. Mugford SG, Yoshimoto N, Reichelt M, Wirtz M, Hill L, Mugford ST, et al. Disruption of adenosine-5′-phosphosulfate kinase in *Arabidopsis* reduces levels of sulfated secondary metabolites. The Plant Cell. 2009;21(3):910-27.

12. He P, Shan L, Lin N-C, Martin GB, Kemmerling B, Nürnberger T, et al. Specific bacterial suppressors of MAMP signaling upstream of MAPKKK in *Arabidopsis* innate immunity. Cell. 2006;125(3):563-75.
