# Supplementary figures and images for: Albugo-imposed changes to tryptophan-derived antimicrobial metabolite biosynthesis may contribute to suppression of non-host resistance to Phytophthora infestans in Arabidopsis thaliana
Source: BMC Biol. 2017 Mar 20;15:20. doi: 10.1186/s12915-017-0360-z (PMC5358052; doi:10.1186/s12915-017-0360-z)

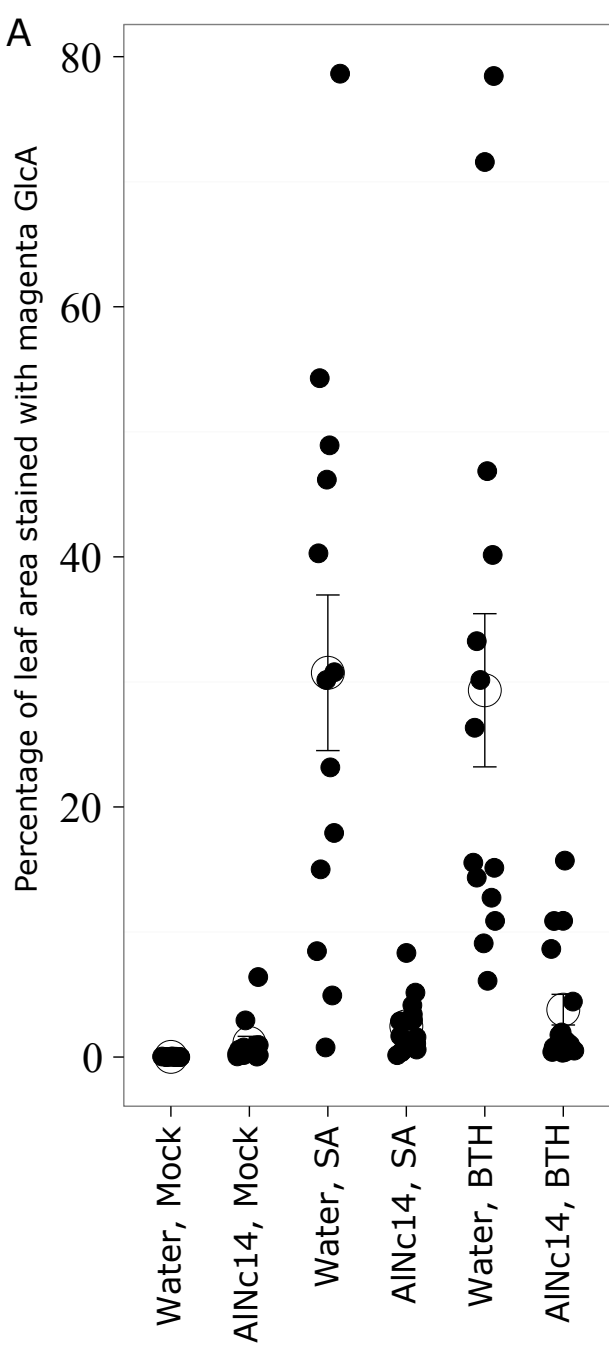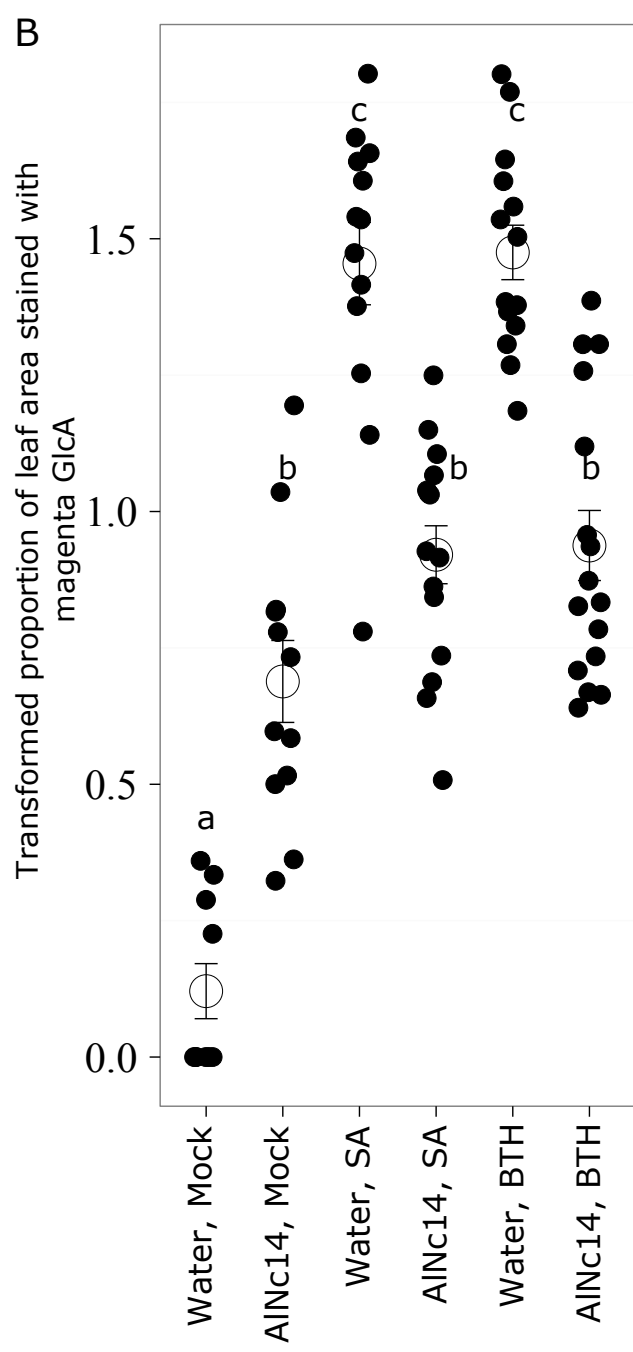

Supplement: Additional file 5: — AlNc14 suppresses benzo-(1,2,3)-thiadiazole-7-carbothioic acid (BTH) and salicylic acid (SA) induction of PR1. To visualize reporter gene induction and pathogen growth in the same leaf, leaves were collected and stained with magenta-GUS to reveal GUS activity, followed by trypan blue to reveal pathogen growth. Leaves of Col-0 pro(PR1)::GUS were previously inoculated with water or AlNc14 (13 dpi) and infiltrated with DMSO (mock), BTH (200 μM) or SA (200 μM) for 8 hours, then stained and examined with a microscope. The percentage of each leaf stained with GUS was determined using ImageJ. (A) Open circles represent mean ± SE of the raw data (percentage of leaf stained) of three independent biological replicates with between two and seven technical replicates per biological replicate (bars left to right n = 10, 12, 13, 15, 14 and 16). (B) Open circles represent mean ± SE of the transformed data (arcsine square root transformation followed by log10 transformation) of three independent biological replicates with between two and seven technical replicates per biological replicate (bars left to right n = 10, 12, 13, 15, 14 and 16). Different letters indicate significant differences P < 0.001 (Two-way ANOVA, Tukey’s HSD test). (PDF 33 kb) [file 12915_2017_360_MOESM5_ESM.pdf]

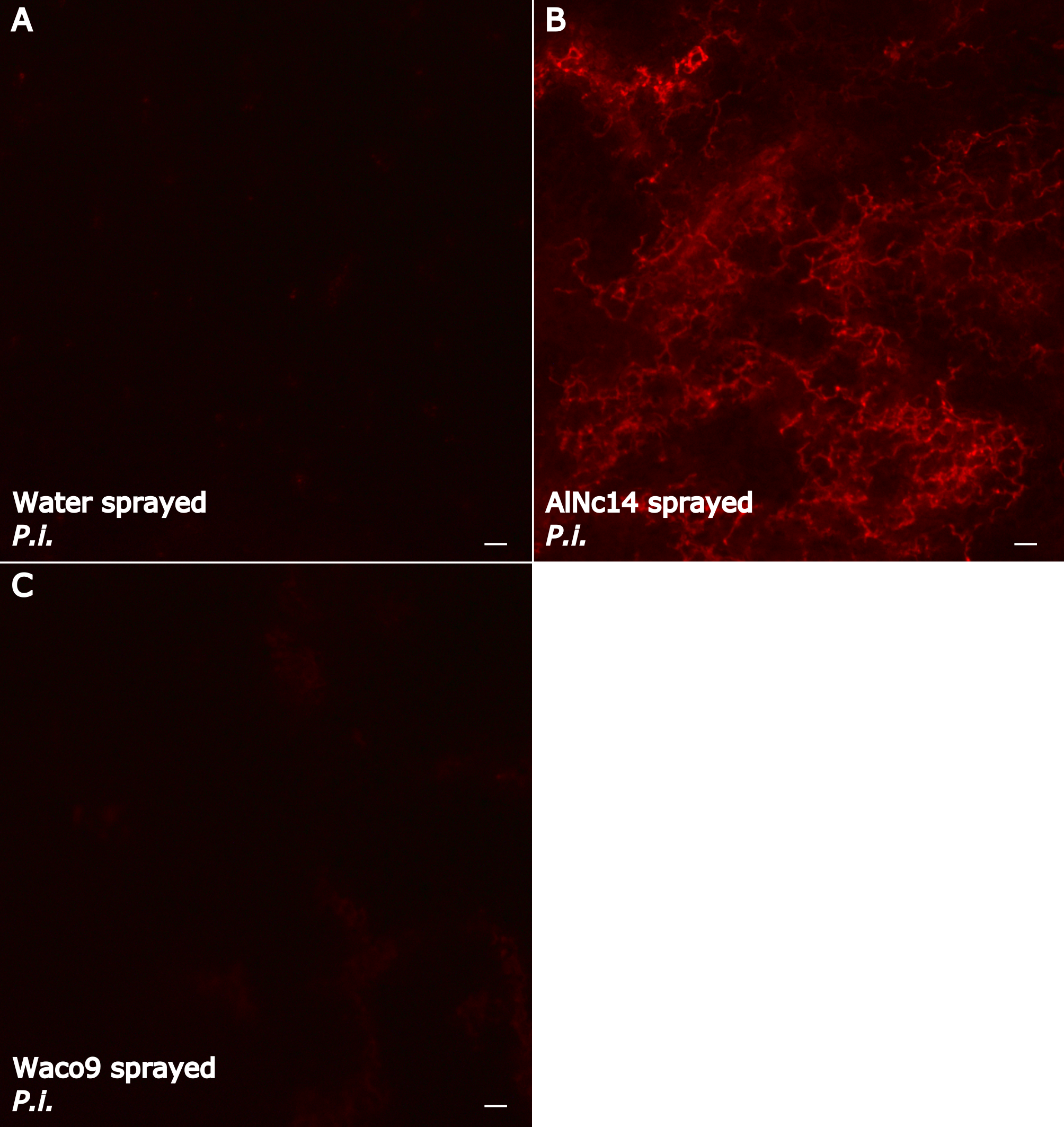

Supplement: Additional file 6: — Hyaloperonospora arabidopsidis (Hpa) Waco9 infection does not allow P. infestans colonization of Arabidopsis. (A) Water sprayed, (B) AlNc14 sprayed (12 dpi) and (C) Hpa sprayed leaves (6 dpi) were drop inoculated with 100 μL of 3.25 to 5 × 10^4^ spores per mL P. infestans 88069td. Fluorescence microscopy of the adaxial surface of the leaf taken at 3 dpi P. infestans. Scale bar: 100 μm. Results shown are representative of two independent experiments. (TIF 3605 kb) [file 12915_2017_360_MOESM6_ESM.tif]

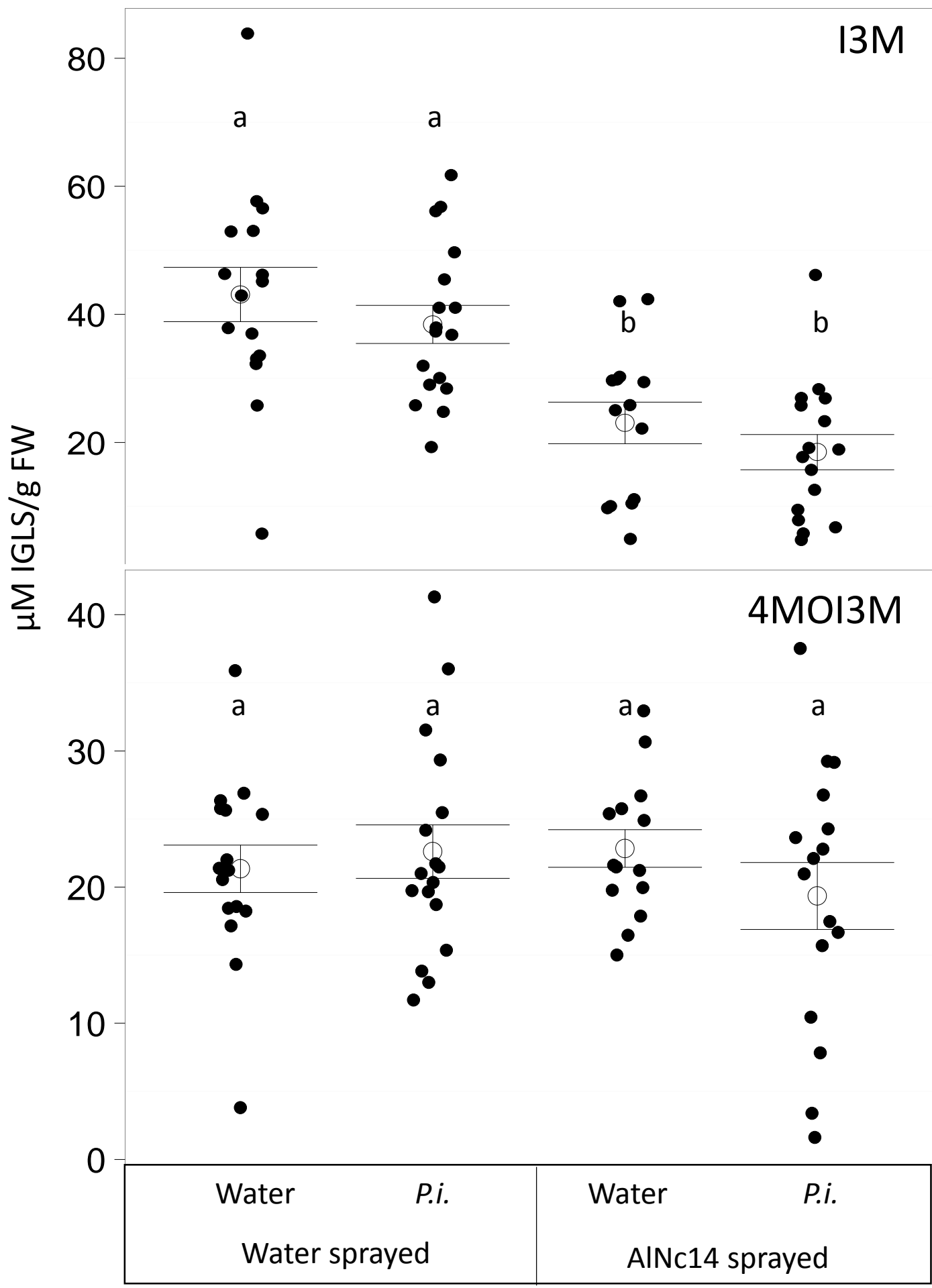

Supplement: Additional file 12: — Indolic glucosinolate measurements in pen2-1 plants in response to pre-treatment with water or Albugo and subsequent inoculation with water or P. infestans. HPLC analysis of mock or Albugo infected pen2-1 tissue (12 dpi), 20 hours post mock or P. infestans treatment (100 μL of 3 × 10^5^ spores per mL). Open circles and bars denote mean indolic glucosinolate content ± SE of three independent biological replicates with six technical replicate per biological replicate. Closed, black circles denote the individual data points. Different letters indicate significant different values within each glucosinolate measured (P < 0.05) (Two-way ANOVA, Tukey’s HSD test). (PDF 175 kb) [file 12915_2017_360_MOESM12_ESM.pdf]

RFP

Brightfield

Overlay

A

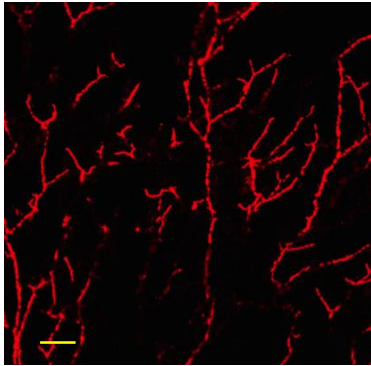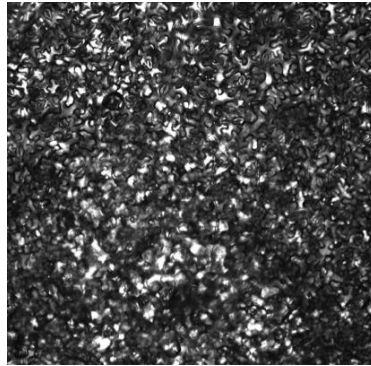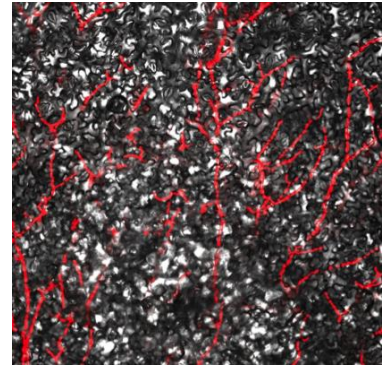

B

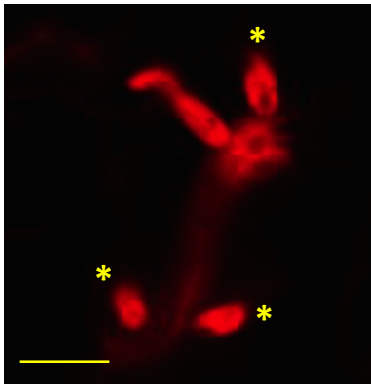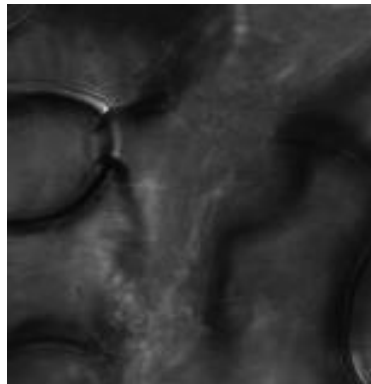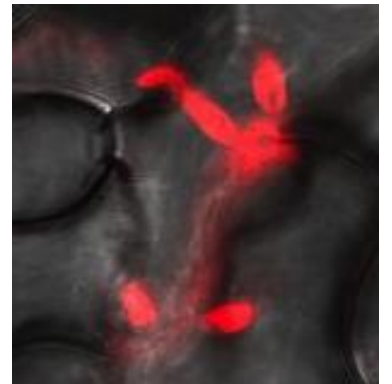

C

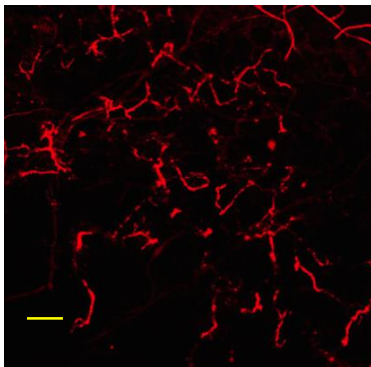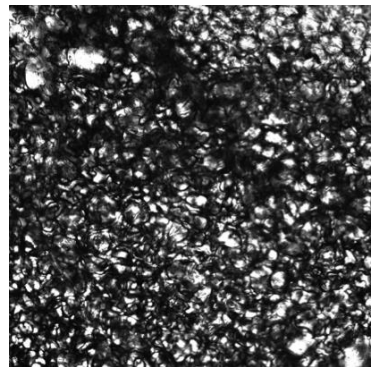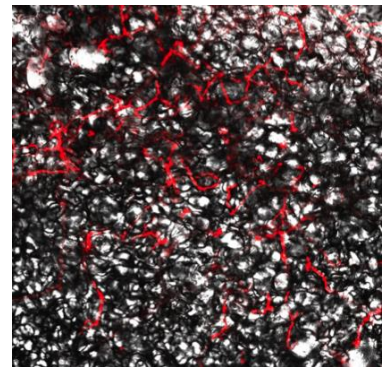

D

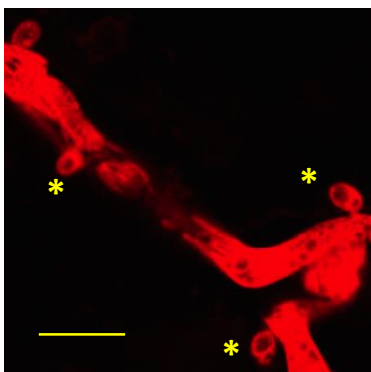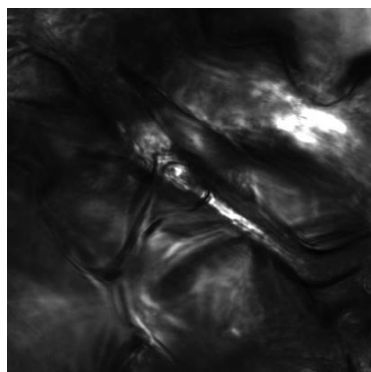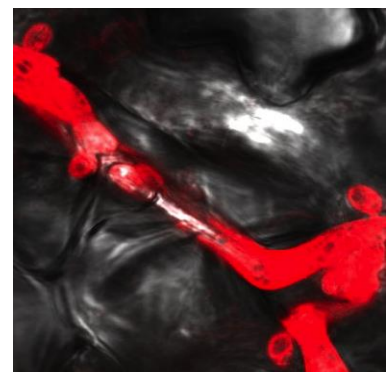

Supplement: Additional file 14: — P. infestans forms haustoria in cyp79b2/b3 plants. Leaves of Nicotiana benthamiana (A and B) and Arabidopsis cyp79b2/b3 (C and D) were drop inoculated with 50 μL of 1 × 10^5^ spores per mL P. infestans 88069td and examined using confocal microscopy at 2 dpi (A and B) and 3 dpi (C and D). A and C show colonization of the leaf by P. infestans. Scale bar = 100 μM. B and D show formation of infection structures by P. infestans, with haustoria denoted by asterisks. Scale bar = 10 μM. (PDF 485 kb) [file 12915_2017_360_MOESM14_ESM.pdf]

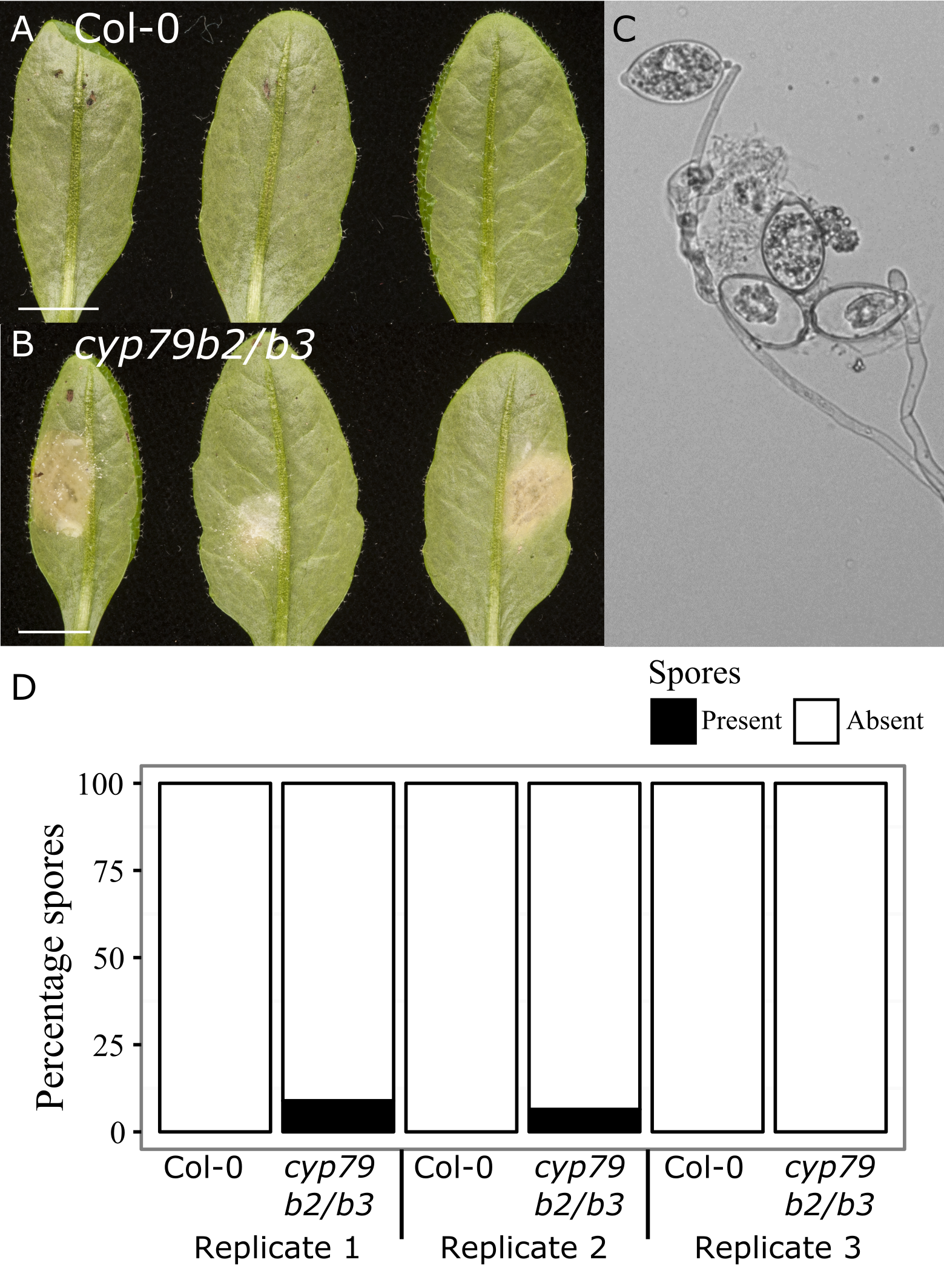

Supplement: Additional file 15: — P. infestans sporulation on cyp79b2/b3 plants. Leaves of Col-0 and cyp79b2/b3 were inoculated with 100 μL of 2.5 × 10^5^ spores per mL P. infestans NL12226. Photographs were taken of the abaxial surface of Col-0 (A) and cyp79b2/b3 (B) leaves at 3 dpi. Scale bars: 5 mm. (C and D) Leaves were examined for sporulation between 3 and 5 dpi by placing water droplets on the leaves and examining them for the presence of spores using a light microscope (C). Frequency of sporulating leaves in three independent experiments were recorded (D) (Replicate 1: Col-0, n = 44, sporulating = 0; cyp79b2/b3, n = 56, sporulating = 5. Replicate 2: Col-0, n = 42, sporulating = 0; cyp79b2/b3, n = 62, sporulating = 4. Replicate 3: Col-0, n = 22, sporulating = 0; cyp79b2/b3, n = 71, sporulating = 0). (TIF 9710 kb) [file 12915_2017_360_MOESM15_ESM.tif]

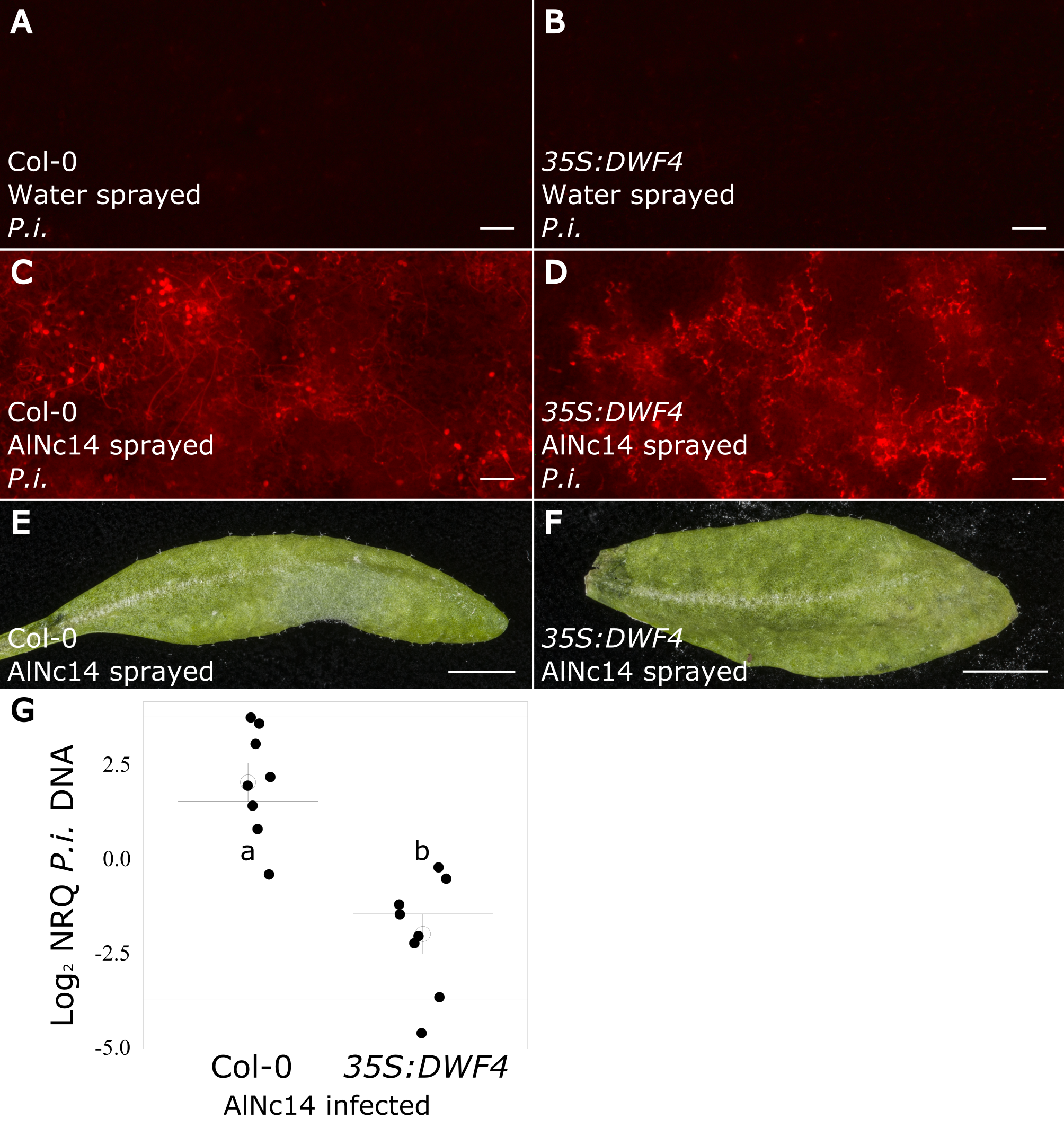

Supplement: Additional file 16: — Albugo-infected 35S:DWF4 is less susceptible to P. infestans than Albugo-infected Col-0. (A–D) Fluorescence microscopy of the adaxial surface of water-sprayed Col-0 (A), water-sprayed 35S:DWF4 (B), Albugo-infected Col-0 (C) and Albugo-infected 35S:DWF4 (D) leaves. Leaves were sprayed with water or Albugo and subsequently inoculated (12 days post spraying) with 100 μL of 1 × 10^5^ spores per mL P. infestans 88069td. Leaves were examined using fluorescence microscopy at 3 dpi. Red fluorescence denotes P.i. growth. Scale bars: 200 μm. Results shown are representative of three independent experiments. (E and F) Photographs of Albugo-infected Col-0 (E) and Albugo-infected 35S:DWF4 (F) leaves, infected as described above, were taken at 3 dpi. Scale bars: 5 mm. (G) Quantification of P. infestans biomass on Albugo infected Col-0 and 35S:DWF4 by qRT-PCR. Leaves were inoculated with 100 μL of 1 × 10^5^ spores per mL P. infestans 88069td. DNA was extracted at 3 dpi and the proportion of P. infestans DNA to plant DNA determined using qRT-PCR. Open circles and bars denote means ± SE of three independent biological replicates with three technical replicates per biological replicate. Closed, black circles denote the individual data points. Different letters indicate significant differences (Welch two sample t-test) (P < 0.001). (TIF 3282 kb) [file 12915_2017_360_MOESM16_ESM.tif]

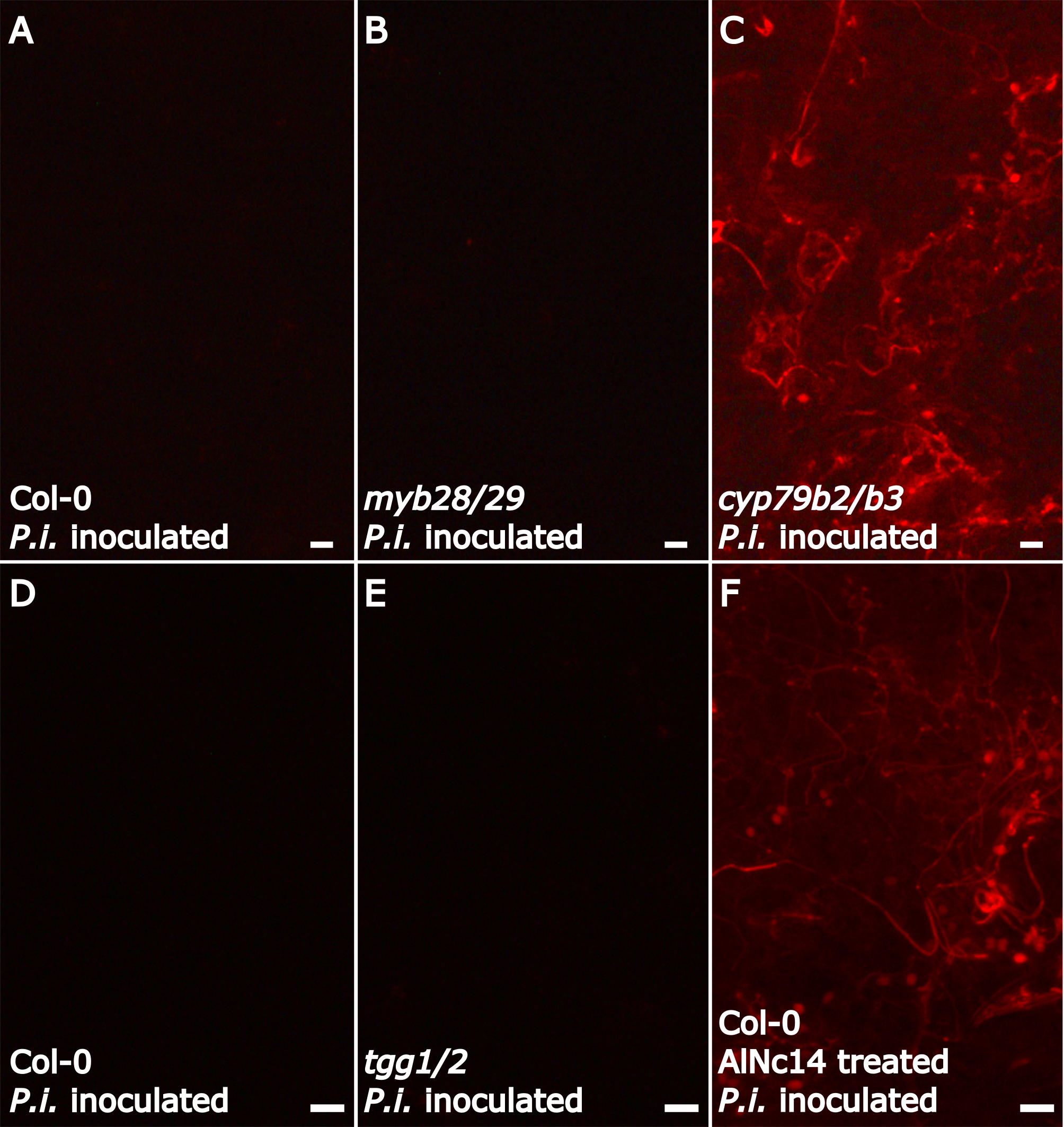

Supplement: Additional file 18: — myb28/29 and tgg1/tgg2 are not susceptible to P. infestans. (A–C) Leaves of Col-0, myb28/29 and cyp79b2/b3 (positive control) were inoculated with 100 μL of at least 1 × 10^5^ spores per mL P. infestans 88069td. (D–F) Leaves of Col-0, tgg1/2 and AlNc14 sprayed Col-0 (positive control) were inoculated with 100 μL of at least 1 × 10^5^ spores per mL P. infestans 88069td. The adaxial surface of the leaves was examined using fluorescence microscopy at 3 dpi. Scale bars: 100 μm. Red fluorescence denotes P.i. growth. Results shown are representative of two (A–C) and three (D–F) independent experiments. (TIF 5249 kb) [file 12915_2017_360_MOESM18_ESM.tif]

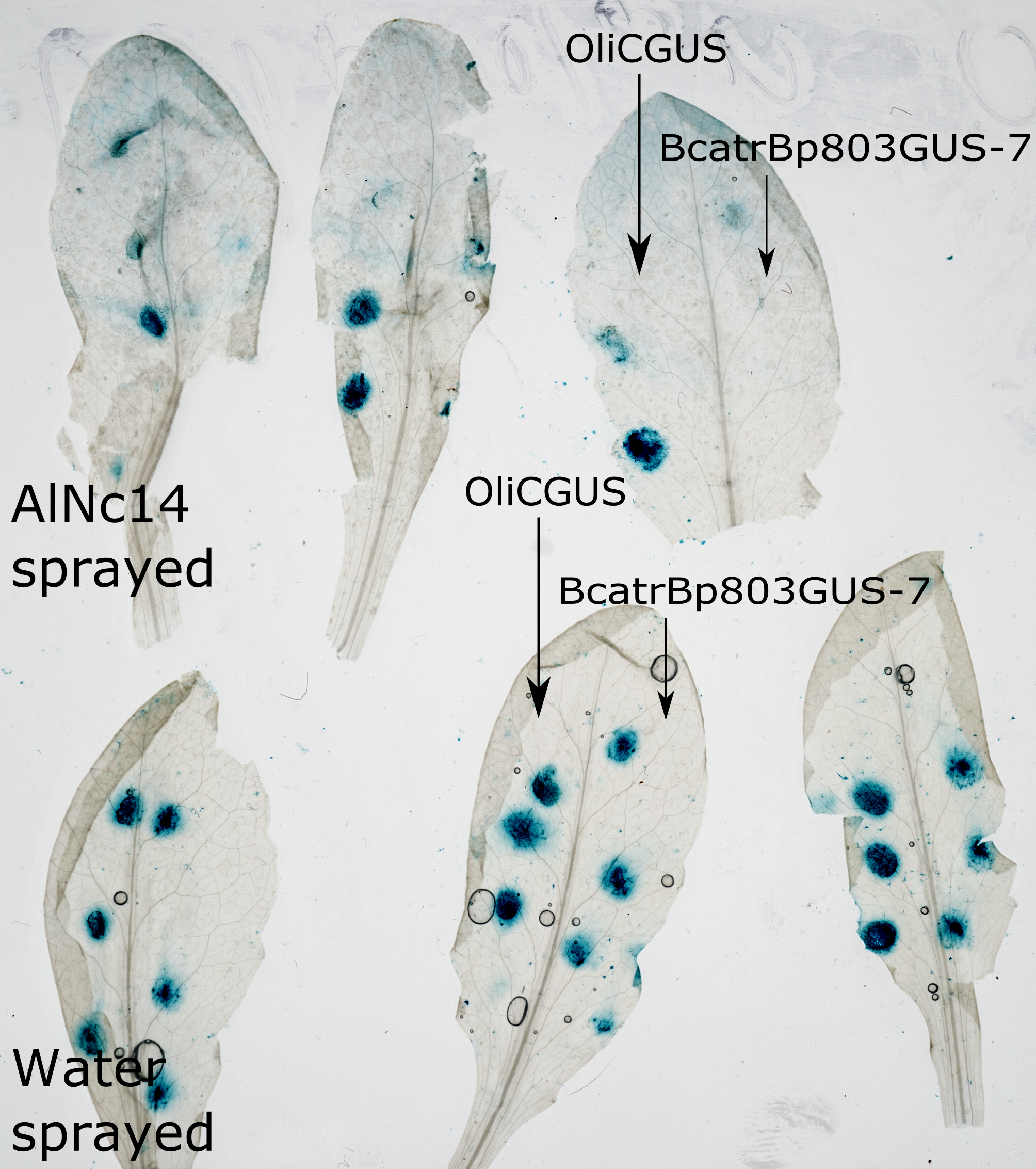

Supplement: Additional file 20: — Example of staining of GUS-expressing B. cinerea strains on water and Albugo-sprayed Col-0 leaves. Photograph of three representative leaves. Top row are AlNc14-infected leaves and the bottom row are water-sprayed leaves. The left hand side of each leaf received three droplets of OliCGUS B. cinerea and the right hand side received three drops of BcatrBp803GUS-7 B. cinerea. Leaves were removed from the plant and stained at 2 dpi B. cinerea. (TIF 6351 kb) [file 12915_2017_360_MOESM20_ESM.tif]
