# Supplementary material for: Albugo-imposed changes to tryptophan-derived antimicrobial metabolite biosynthesis may contribute to suppression of non-host resistance to Phytophthora infestans in Arabidopsis thaliana
Source: BMC Biol. 2017 Mar 20;15:20. doi: 10.1186/s12915-017-0360-z (PMC5358052; doi:10.1186/s12915-017-0360-z)
Supplement: Additional file 9: — Two-way ANOVA results from qRT-PCR of tryptophan-derived secondary metabolite genes at 6 hours post P. infestans inoculation. ANOVA table. (DOCX 13 kb) [file 12915_2017_360_MOESM9_ESM.docx]

**Additional file 8. Two-way ANOVA results from qRT-PCR of tryptophan-derived secondary metabolite genes at 6 hours post *P. infestans* inoculation.**

| **Gene** | **Source of variation** | **Degrees of freedom** | **Sum of Squares** | **Mean squares** | ***F*-value** | ***P*-value** |
| --- | --- | --- | --- | --- | --- | --- |
| *CYP71A13* | Pre-treatment | 1 | 13.37 | 13.37 | 16.53 | 0.000603 |
|  | Inoculation | 1 | 192.46 | 192.46 | 237.93 | 1.44e-12 |
|  | Pre-treatment*Inoculation | 1 | 41.32 | 41.32 | 51.08 | 6.36e-07 |
|  | Residuals | 20 | 16.18 | 0.81 |  |  |
| *PAD3* | Pre-treatment | 1 | 0 | 0 | 0 | 0.990343 |
|  | Inoculation | 1 | 143.27 | 143.27 | 115.17 | 9.52e-10 |
|  | Pre-treatment*Inoculation | 1 | 28.38 | 28.38 | 22.82 | 0.000115 |
|  | Residuals | 20 | 24.88 | 1.24 |  |  |
| *CYP79B2* | Pre-treatment | 1 | 0.21 | 0.21 | 0.129 | 0.7234 |
|  | Inoculation | 1 | 55.29 | 55.29 | 33.557 | 1.14e-05 |
|  | Pre-treatment*Inoculation | 1 | 8.85 | 8.85 | 5.374 | 0.0311 |
|  | Residuals | 20 | 32.95 | 1.65 |  |  |
| *CYP83B1* | Pre-treatment | 1 | 0.01 | 0.005 | 0.001 | 0.976 |
|  | Inoculation | 1 | 2.56 | 2.56 | 0.444 | 0.513 |
|  | Pre-treatment*Inoculation | 1 | 0.25 | 0.254 | 0.044 | 0.836 |
|  | Residuals | 20 | 115.38 | 5.769 |  |  |
| *SOT16* | Pre-treatment | 1 | 2.617 | 2.617 | 5.249 | 0.032956 |
|  | Inoculation | 1 | 10.066 | 10.066 | 20.185 | 0.000223 |
|  | Pre-treatment*Inoculation | 1 | 1.608 | 1.608 | 3.225 | 0.087646 |
|  | Residuals | 20 | 9.974 | 0.499 |  |  |
| *CYP81F2* | Pre-treatment | 1 | 11.43 | 11.43 | 11.97 | 0.002478 |
|  | Inoculation | 1 | 81.88 | 81.88 | 85.73 | 1.14e-08 |
|  | Pre-treatment*Inoculation | 1 | 20.05 | 20.05 | 20.99 | 0.000181 |
|  | Residuals | 20 | 19.10 | 0.96 |  |  |
