# Supplementary material for: Albugo-imposed changes to tryptophan-derived antimicrobial metabolite biosynthesis may contribute to suppression of non-host resistance to Phytophthora infestans in Arabidopsis thaliana
Source: BMC Biol. 2017 Mar 20;15:20. doi: 10.1186/s12915-017-0360-z (PMC5358052; doi:10.1186/s12915-017-0360-z)
Supplement: Additional file 11: — Two-way ANOVA results from qRT-PCR of tryptophan-derived secondary metabolite genes at 48 hours post P. infestans inoculation. ANOVA table. (DOCX 13 kb) [file 12915_2017_360_MOESM11_ESM.docx]

**Additional file 10. Two-way ANOVA results from qRT-PCR of tryptophan-derived secondary metabolite genes at 48 hours post *P. infestans* inoculation.**

| **Gene** | **Source of variation** | **Degrees of freedom** | **Sum of Squares** | **Mean squares** | ***F*-value** | ***P*-value** |
| --- | --- | --- | --- | --- | --- | --- |
| *CYP71A13* | Pre-treatment | 1 | 17.71 | 17.71 | 15.788 | 0.00081 |
|  | Inoculation | 1 | 84.43 | 84.43 | 75.266 | 4.93e-08 |
|  | Pre-treatment*Inoculation | 1 | 4.69 | 4.69 | 4.179 | 0.05504 |
|  | Residuals | 19 | 21.31 | 1.12 |  |  |
| *PAD3* | Pre-treatment | 1 | 7.51 | 7.51 | 8.103 | 0.0103 |
|  | Inoculation | 1 | 63.44 | 63.44 | 68.455 | 1.01e-07 |
|  | Pre-treatment*Inoculation | 1 | 1.04 | 1.04 | 1.12 | 0.3031 |
|  | Residuals | 19 | 17.61 | 0.93 |  |  |
| *CYP79B2* | Pre-treatment | 1 | 10.559 | 10.559 | 23.283 | 0.000118 |
|  | Inoculation | 1 | 26.967 | 26.967 | 59.461 | 2.88e-07 |
|  | Pre-treatment*Inoculation | 1 | 0.711 | 0.711 | 1.568 | 0.225732 |
|  | Residuals | 19 | 8.617 | 0.454 |  |  |
| *CYP83B1* | Pre-treatment | 1 | 13.615 | 13.615 | 15.808 | 0.000744 |
|  | Inoculation | 1 | 0.513 | 0.513 | 0.595 | 0.449353 |
|  | Pre-treatment*Inoculation | 1 | 2.436 | 2.436 | 2.829 | 0.108137 |
|  | Residuals | 19 | 17.225 | 0.861 |  |  |
| *SOT16* | Pre-treatment | 1 | 1.413 | 1.413 | 2.333 | 0.143 |
|  | Inoculation | 1 | 0.278 | 0.278 | 0.459 | 0.506 |
|  | Pre-treatment*Inoculation | 1 | 0.189 | 0.189 | 0.312 | 0.583 |
|  | Residuals | 19 | 11.509 | 0.6057 |  |  |
| *CYP81F2* | Pre-treatment | 1 | 39.12 | 39.12 | 45.313 | 1.97e-06 |
|  | Inoculation | 1 | 22.33 | 22.33 | 25.870 | 6.56e-05 |
|  | Pre-treatment*Inoculation | 1 | 3.67 | 3.67 | 4.256 | 0.0531 |
|  | Residuals | 19 | 16.40 | 0.86 |  |  |
