# Supplementary material for: Albugo-imposed changes to tryptophan-derived antimicrobial metabolite biosynthesis may contribute to suppression of non-host resistance to Phytophthora infestans in Arabidopsis thaliana
Source: BMC Biol. 2017 Mar 20;15:20. doi: 10.1186/s12915-017-0360-z (PMC5358052; doi:10.1186/s12915-017-0360-z)
Supplement: Additional file 21: — B. cinerea detects less available camalexin in Albugo-infected tissue. Leaves underwent protein extraction and GUS enzyme activity was determined using a fluorescence-based assay. Results were normalized to B. cinerea weight proportion of each sample using qRT-PCR on Botrytis and Arabidopsis genomic DNA. Open circles and bar dots represent the mean ± SE of three independent biological replicates with three or four technical replicates per biological replicate. Closed, black circles denote the individual data points. Asterisk indicates significant differences measured at P < 0.05 (Wilcoxon rank sum test within B. cinerea strain followed by Bonferroni correction), n.s. = not significant. (PDF 176 kb) [file 12915_2017_360_MOESM21_ESM.pdf]

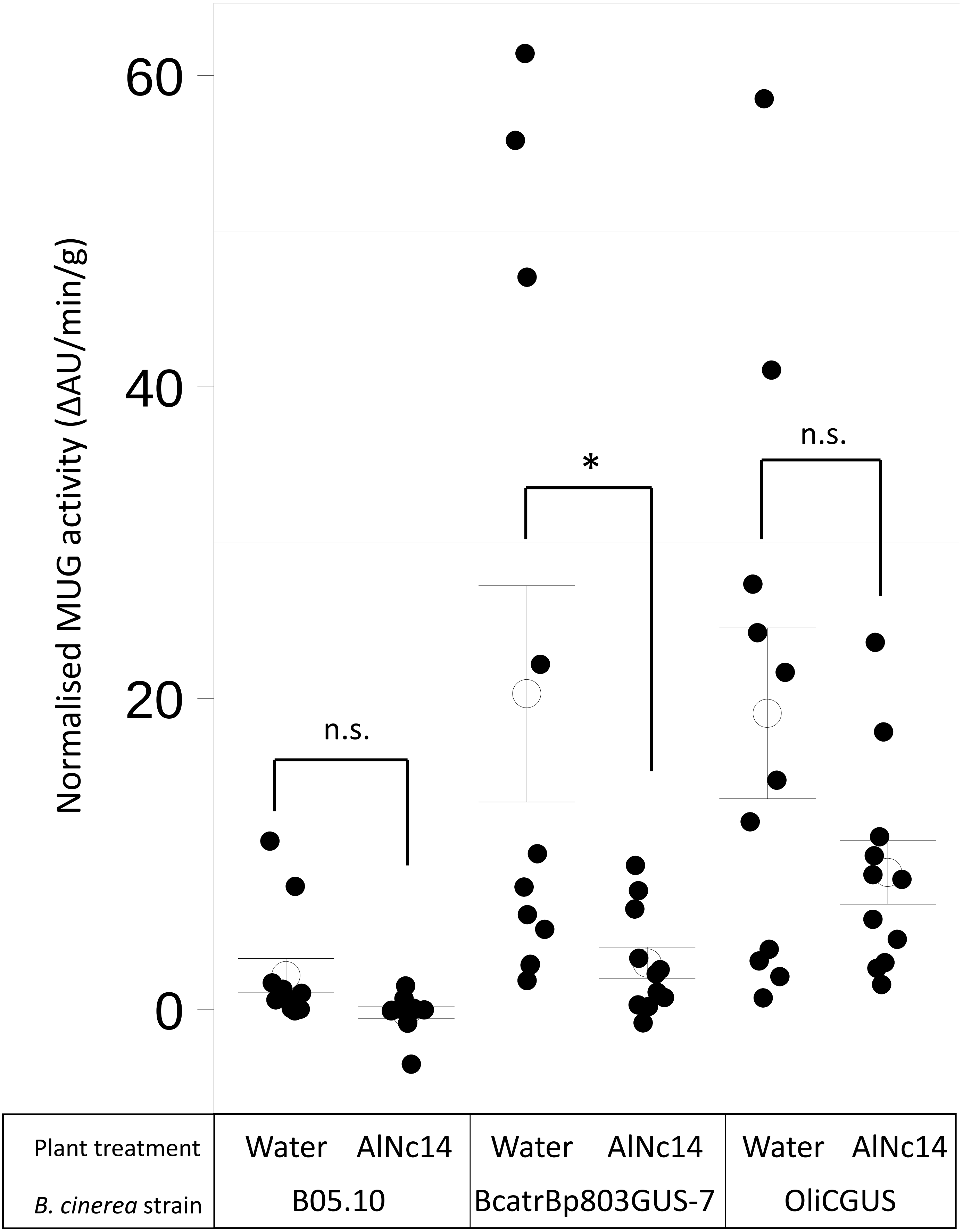

Normalised MUG activity (ΔAU/min/g)
60
40
20
0
n.s.
*
n.s.
Plant treatment
Water
AlNc14
Water
AlNc14
Water
AlNc14
B. cinerea strain
B05.10
BcatrBp803GUS-7
OliCGUS
