# Supplementary material for: Albugo-imposed changes to tryptophan-derived antimicrobial metabolite biosynthesis may contribute to suppression of non-host resistance to Phytophthora infestans in Arabidopsis thaliana
Source: BMC Biol. 2017 Mar 20;15:20. doi: 10.1186/s12915-017-0360-z (PMC5358052; doi:10.1186/s12915-017-0360-z)
Supplement: Additional file 23: — List of selected, lower level gene ontology (GO) terms enriched in genes differentially expressed during AlNc14 infection but not AcNc2 infection. Table showing the GO terms enriched in Arabidopsis genes differentially expressed during AlNc14 infection only. (DOCX 14 kb) [file 12915_2017_360_MOESM23_ESM.docx]

Additional file 22. List of selected, lower level GO terms enriched in genes differentially expressed during AlNc14 infection but not AcNc2 infection.

| **Category** | **2 dpi** | **4 dpi** | **6 dpi** | **8 dpi** | **Combined time points** |
| --- | --- | --- | --- | --- | --- |
| Up-regulated vs. control (0 dpi) | - No significant terms | - RNA processing | - Glucosinolate metabolic process - Response to hormone stimulus - Response to sucrose stimulus - Response to UV-B | - Anthocyanin biosynthesis - Response to hormone stimulus - Response to sucrose stimulus - Response to UV-B | - Response to hormone stimulus - Response to sucrose stimulus - Response to UV-B |
| Down-regulated vs. control (0 dpi) | - Too few genes to analyse | - No significant terms | - Defense response to bacterium - Defense response to fungus - MAPKKK cascade - Photosynthesis - RNA elongation - Salicylic acid biosynthesis - Salicylic acid mediated signalling pathway - Systemic acquired resistance | - Defense response to bacterium - Defense response to fungus - Jasmonic acid mediated signalling - MAPKKK cascade - RNA elongation - Salicylic acid biosynthesis - Salicylic acid mediated signaling pathway - Systemic acquired resistance | - Defense response to bacterium - Defense response to fungus - Jasmonic acid mediated signalling - MAPKKK cascade - Regulation of plant-type hypersensitive response - Salicylic acid biosynthesis - Salicylic acid mediated signaling pathway - Systemic acquired resistance |
